# Modeling Phenotypic Metabolic Adaptations of *Mycobacterium tuberculosis* H37Rv under Hypoxia

**DOI:** 10.1371/journal.pcbi.1002688

**Published:** 2012-09-13

**Authors:** Xin Fang, Anders Wallqvist, Jaques Reifman

**Affiliations:** DoD Biotechnology High-Performance-Computing Software Applications Institute, Telemedicine and Advanced Technology Research Center, U.S. Army Medical Research and Materiel Command, Fort Detrick, Maryland, United States of America; University of Texas at Austin, United States of America

## Abstract

The ability to adapt to different conditions is key for *Mycobacterium tuberculosis*, the causative agent of tuberculosis (TB), to successfully infect human hosts. Adaptations allow the organism to evade the host immune responses during acute infections and persist for an extended period of time during the latent infectious stage. In latently infected individuals, estimated to include one-third of the human population, the organism exists in a variety of metabolic states, which impedes the development of a simple strategy for controlling or eradicating this disease. Direct knowledge of the metabolic states of *M. tuberculosis* in patients would aid in the management of the disease as well as in forming the basis for developing new drugs and designing more efficacious drug cocktails. Here, we propose an *in silico* approach to create state-specific models based on readily available gene expression data. The coupling of differential gene expression data with a metabolic network model allowed us to characterize the metabolic adaptations of *M. tuberculosis* H37Rv to hypoxia. Given the microarray data for the alterations in gene expression, our model predicted reduced oxygen uptake, ATP production changes, and a global change from an oxidative to a reductive tricarboxylic acid (TCA) program. Alterations in the biomass composition indicated an increase in the cell wall metabolites required for cell-wall growth, as well as heightened accumulation of triacylglycerol in preparation for a low-nutrient, low metabolic activity life style. In contrast, the gene expression program in the deletion mutant of *dosR*, which encodes the immediate hypoxic response regulator, failed to adapt to low-oxygen stress. Our predictions were compatible with recent experimental observations of *M. tuberculosis* activity under hypoxic and anaerobic conditions. Importantly, alterations in the flow and accumulation of a particular metabolite were not necessarily directly linked to differential gene expression of the enzymes catalyzing the related metabolic reactions.

## Author Summary

*Mycobacterium tuberculosis* latently infects one-third of the human population and is responsible for millions of deaths worldwide every year. The ability of the pathogen to persist in the human population stems from its capacity to adapt to host-induced stresses and adjust its metabolism to different host environments. We have developed a novel model to interpret *M. tuberculosis* H37Rv metabolic adjustment by combining gene transcription data with a genome-scale metabolic network model. Using our model, we were able to identify the changes in the metabolic program associated with hypoxia, predict phenotypic change, and determine the critical metabolic enzymes and pathways that are required for pathogen survival. In particular, we predicted the switch in the tricarboxylic acid cycle from an oxidative to a reductive path. The altered importance of different metabolites and pathways under hypoxic conditions may provide guidance for designing novel, adjuvant drug therapies for clearing persistent and latent infections.

## Introduction

*Mycobacterium tuberculosis*, the causative agent of tuberculosis (TB), caused 8.8 million new TB cases and resulted in the death of 1.5 million people worldwide in 2010 [1]. Furthermore, it is estimated that one-third of the human population is latently infected with the disease, with an overall lifetime risk of developing active TB disease of 10% [2]. In the United States, more than 80% of clinically observed TB results from reactivated latent infections [3], [4]. The latent disease state prevents eradication, confounds diagnosis, increases HIV comorbidity [5], prolongs existing TB treatment to at least six months [6], [7], and increases the risk for the development of drug resistance [8]. The variety of disease states, ranging from dormant to sub-clinical to clinical disease manifestations, complicates the treatment and eradication of the disease [9]. The presence of latent infections results in a dangerous reservoir of the disease. The manifold of latent disease manifestations [10], [11] is poorly understood and difficult to replicate in model systems of TB, making it quite challenging to reach the United Nation's goal of eradicating TB before 2050 [12] and developing effective therapeutics [13].

Targeting different aspects of metabolism in the latent state is a viable therapeutic strategy that is supported by evidence of differential metabolic activity among several dormant and latent states of *M. tuberculosis* [14]–[17]. One of the challenges in targeting metabolism in latent disease is the inability of existing experimental model systems to fully capture the range of observed phenotypes. While experimental *in vitro* persister models can be created based on acid stress, hypoxic stress, and carbon starvation [18], there is a need for studying the manifold of disease states. Here, we propose an *in silico* approach to create state-specific models based on readily available gene expression data. The coupling of differential gene expression data with a metabolic network model allows us to metabolically characterize any TB disease state, provided the corresponding microarray data are available. We applied this technique to characterize the metabolic adaptations of *M. tuberculosis* in response to hypoxia.

Similar to the introduction of nitric oxide [19] and carbon monoxide [20], hypoxia is one of the factors that characterize the onset of persistence and latency in *M. tuberculosis* [21]. Although hypoxic microenvironments are an important feature of tuberculosis granulomas in guinea pig, rabbit, and nonhuman primate models of the disease, this feature is not present in mouse models [22], pointing to a link between host-specific factors and latency. In addition to these models, there are established protocols to cultivate the pathogen in artificial low-oxygen conditions in order to create *in vitro* persistence models [23]. These models, characterized by low-oxygen conditions, exhibit gene expression profiles distinct from those obtained under normoxic conditions [24]–[28]. The immediate response to hypoxic stress is partially governed by the *dosR* gene, which encodes a transcription factor essential for the hypoxic persistence of mycobacteria [29]. In particular, Park and coworkers measured changes in gene expression under hypoxia of wild type *M. tuberculosis* H37Rv and its Δ*dosR* deletion mutant compared to normoxia [25]. Although this work established the connection of the *dosR* regulator to the hypoxic response, reviewing the list of differentially expressed genes provides a limited view of what the *dosR*-initiated gene expression pattern entails in terms of *M. tuberculosis* metabolic adaptation to hypoxia.

A systems-level understanding of metabolism requires the identification and reassembly of the constituent components (metabolites, reactions, transport, and uptake processes) and methods to analyze metabolic phenotypes [30]–[34]. The most robust and advanced systems biology reconstruction and analysis techniques focus on metabolism. In particular, genome-scale metabolic networks for *M. tuberculosis* are composed of hundreds of distinct but interconnected chemical reactions, each processing particular metabolites that, taken together, ultimately allow the cell to function and grow [35], [36]. Metabolic network reconstructions of *M. tuberculosis* have been used to identify genes essential for growth [35], [36], study the importance of mycolic acid production [37], model quantitative drug-dose response [38], [39], deconstruct metabolic responses [40], and identify metabolic adaptations to different *in vitro*, *ex vivo*, and *in vivo* host conditions [41], [42]. Traditional metabolic network analysis results in a general description of a cell's steady state metabolism and typically represents an idealized version of the cellular metabolic program under exponential growth conditions. As such, the network description does not take into account different protein or expression levels of individual metabolic genes in the network.

Gene expression data captures the transcriptional state of a cell in a particular biological state and it is challenging to interpret this partial information with respect to an altered metabolic program. The strength of a transcriptional approach is that we can capture a specific snapshot of the cell without elaborating the underlying signaling and gene regulatory networks. The weakness is that the transcriptional state is not a direct readout of the metabolic enzyme concentrations that perform metabolic reactions. Efforts to connect transcriptional levels to metabolic activity in network models of metabolism have focused on correlating absolute expression levels to metabolite flows [37], [43], [44], completely suppressing reactions based on pre-defined changes in relative expression levels [45], or establishing protocols for generating condition-specific metabolic signals of changes in metabolite production based on multiple microarray data sets [40]. Here, we introduce a new method that relies on relative gene expression levels between a metabolically well-characterized reference state (e.g., exponential growth under normoxic conditions) and a perturbed state of interest (e.g., reduced growth under hypoxic conditions). Although this method ultimately relies on a correlation between gene transcription levels and enzymatic activity, in contrast to previous methods [37], [43], [44], we rely on individual relative relationships between a reference condition and a condition of interest for each gene. It allows for a continuous flow of metabolites, even for down-regulated enzymes, and accommodates variability in biomass composition. This latter feature overcomes the restriction of constraint-based models that the biomass composition remains fixed under the studied conditions, as biomass variability occurs for several bacterial species under different growth conditions [46]–[48].

Thus, based only on the differential gene expression data from *M. tuberculosis* H37Rv under hypoxic conditions [25], we mapped out the metabolic response to low-oxygen stress. The model correctly predicted lower oxygen uptake, a lowered ATP production rate, and a higher hypoxic growth rate as compared to its Δ*dosR* deletion mutant, indicating that the presence of the *dosR* gene was essential for the pathogen to adapt to hypoxia [49]–[51]. We also predicted that hypoxia induces the production of cell-wall metabolites and alters the biomass composition of *M. tuberculosis* [50], [51]. Importantly, our model indicates that the glucose-processing glycolysis pathway and the reductive side of the tricarboxylic acid (TCA) cycle contribute to the adaptation of *M. tuberculosis* to hypoxia [16], [52] and could serve as a drug target for the elimination of this pathogen in latent disease states.

## Results

### An example network

**Figure 1:**
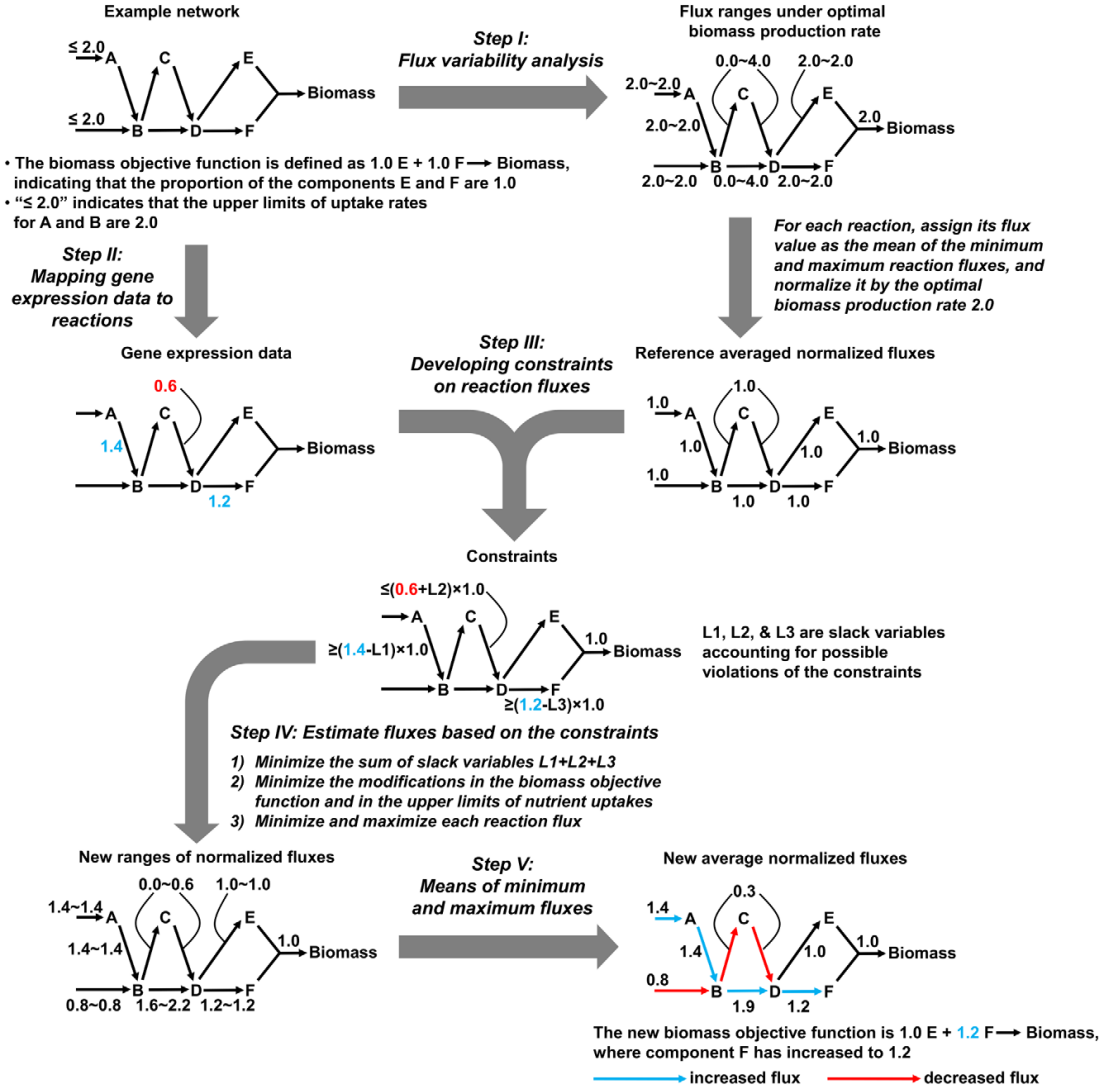
Schematic description of integrating a small example metabolic network with gene expression data. Construction of the altered metabolic state used gene expression data to constrain and alter the reference fluxes obtained from a metabolic network compatible with the reference condition. The example network contained six metabolites (A–F), two uptake reactions, six enzymatic reactions, and one biomass reaction. In the reference condition, the biomass function contained equal amounts of metabolites E and F, set to 1.0 millimoles per gram dry weight of the organism (mmol/gDW), and the uptake rates for the metabolites A and B were each assigned an upper limit of 2.0 mmol/(h·gDW). In Step I, we obtained the minimum and maximum fluxes under the optimal biomass production rate via flux variability analysis and calculated the average normalized flux for the reference metabolic network. In Step II, the gene expression ratios were mapped to their corresponding reactions. In Step III, we initially set constraints for reactions that were associated with altered gene expression values. These constraints were based on the normalized reference network with the biomass production rate set to one and resulted in increased normalized fluxes through reactions related to up-regulated genes (reactions A→B and D→F) and decreased fluxes related to down-regulated genes (reaction C→D). Because biological activities other than gene transcription can influence reaction fluxes, we introduced a set of non-negative slack variables (L1, L2, and L3) to account for possible violations of the constraints. In Step IV, we further performed a number of optimizations subject to the constraints from the previous step and obtained a new minimum and maximum normalized flux for each reaction. We first minimized the overall violation of the developed constraints in the form of the sum of the slack variables (highest priority). We then minimized the modifications in the biomass objective function and those in the upper limits of metabolite uptakes (medium priority), and, last, we minimized and maximized each reaction flux (lowest priority). Finally, in Step V, we constructed the new metabolic state by calculating the new average normalized flux for each reaction as the mean of its new minimum and maximum fluxes. This metabolic state was representative of the new condition and in this case was associated with altered uptake rates, pathway preferences, and an altered biomass composition.

Figure 1 illustrates the integration of microarray data and a metabolic network description for a small example network that contained six metabolites (A–F), two uptake reactions, six enzymatic reactions, and one biomass reaction. In this set, we were given a metabolic network capable of producing biomass for the reference condition and the gene expression ratios for each metabolic reaction between the reference and the new condition. Given relative gene expression ratios, the approach initially constructed a set of normalized relative fluxes for each metabolic reaction in the reference state (Figure 1, Step I) and then introduced the altered gene expression (Figure 1, Step II) as soft constraints on these fluxes (Figure 1, Step III). The soft constraints allowed the system to adjust the flow of metabolites as calculated from the entire network to the given altered gene expression state. The procedure then established new flux ranges for all reactions by minimizing violations of the constraints introduced by the gene expression data, modifications to the biomass, and alterations in the uptake reactions (Figure 1, Step IV). The final metabolic network approximated the altered metabolic state. In the example network, the new condition was compatible with 1) increased (decreased) uptake of metabolite A (B), 2) preferred metabolite flow through reaction B→D over the reaction path B→C→D, 3) increased metabolite flow in reaction B→D even though the gene expression of the enzyme catalyzing this reaction was unchanged, and 4) increased content of metabolite F in the biomass objective function. A detailed description of the procedure is given in the Materials and Methods Section and the rationale for constructing a relative gene expression methodology is further articulated in the Discussion Section. We performed an initial validation of our approach by successfully predicting experimentally measured reaction fluxes from two separate laboratories. These studies examined metabolic fluxes in yeast grown on four different carbon sources [53] and ^13^C flux changes upon removal of the gcn4 global regulator gene under histidine starvation conditions [54] (see Supplemental Text S1).

### Phenotypic metabolic changes of *M. tuberculosis* H37Rv under hypoxia

The persistence of *M. tuberculosis* in human granulomas is partly due to its ability to adapt to a condition of low oxygen availability [23], a process that requires the transcription factor gene *dosR* [19]. We modeled an altered metabolic state of *M. tuberculosis* in response to moving from the reference state of normoxia to an altered hypoxic state as defined by its transcriptional state. We used the *iNJ*661m metabolic network of *M. tuberculosis* H37Rv [42], an enhanced version of the original *iNJ*661 network [35] that retains the correct predictions of growth rates of H37Rv in different media and includes several reactions missed in *iNJ*661, e.g., in the methylcitrate cycle pathway. We integrated this network with microarray data that included gene expression ratios for both induced and repressed mRNA gene transcription for wild type *M. tuberculosis* H37Rv, as well as for the Δ*dosR* deletion mutant, associated with the transfer from normoxic air to hypoxic nitrogen gas with 0.2% oxygen (1.5 mm Hg) [25]. Furthermore, based on experimentally determined normoxic growth [19] and ATP concentrations in culture [49] as well as the fact that the *dosR* gene does not directly encode an enzyme in the metabolic network, we assumed that the normoxic metabolic state was equivalent between the two strains, and thus we used the same network for the normoxic simulation. Using the metabolic network/gene-expression integration model, we predicted hypoxia-induced changes in important phenotypes (oxygen uptake, ATP production, growth), biomass composition, and fluxes through the central carbon metabolism for both wild type *M. tuberculosis* H37Rv and its Δ*dosR* deletion mutant. We used the observed changes in experimental phenotypes to qualitatively validate and indicate the utility of the proposed method [37], [43].

**Figure 2:**
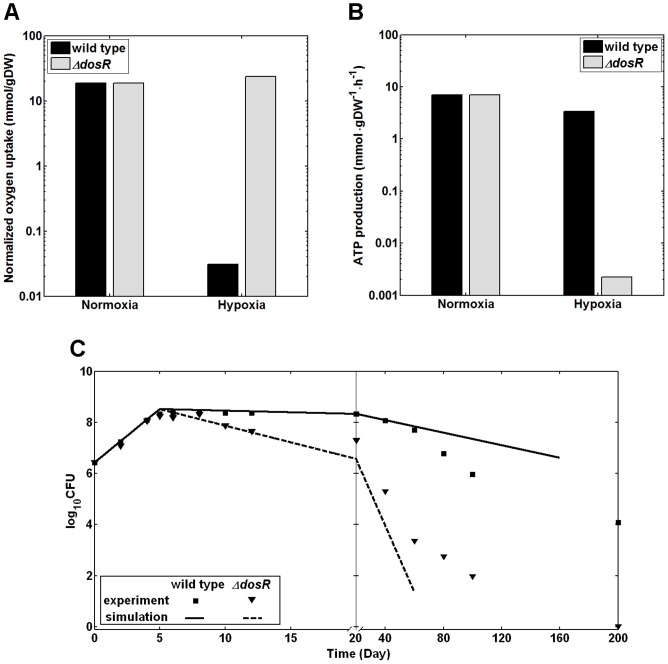
Phenotypic characteristics of wild type *Mycobacterium tuberculosis* H37Rv and Δ*dosR* under normoxia and hypoxia. (A) The predicted normalized oxygen uptake rates of *Mycobacterium tuberculosis* H37Rv and the Δ*dosR* deletion mutant under normoxia and hypoxia. The oxygen uptake rates were normalized by each strain's biomass production rate. Supplemental Table S1 gives the biomass production rates of each strain under different conditions, which were calculated as described in the Materials and Methods Section. We based the metabolic network models of the hypoxic state on differential gene expression data associated with the change from normoxic air to hypoxic nitrogen gas with 0.2% oxygen (1.5 mm Hg) after 2 hours [25]. The wild type metabolic response involved reducing its oxygen requirement to cope with the low-oxygen stress, while the Δ*dosR* deletion mutant was not capable of adjusting. (B) The predicted ATP production levels for the same systems as in panel A showed a slight reduction for the wild type and a much larger decrease for the Δ*dosR* deletion mutant in response to hypoxia. Note that the ATP production rates were not normalized so to facilitate a direct comparison with the experimental data in Refs. [16] and [49]. (C) The modeled growth characteristic of the wild type and Δ*dosR* deletion mutant were compared with the corresponding experimental data [49]. Following the experimental data presentation, the x-axis plots two different time intervals, 0–20 and 20–200 days, using two different time scales. The initial aerobic growth phase for the first 5 days was followed by a slight decrease in cell concentration upon switching to hypoxic conditions on day 5. Our metabolic model interpretation was also compatible with a slight decrease in cell concentration for wild type and a substantial decrease for the deletion mutant. Because the gene expression data was compatible with the immediate hypoxic response, the validity range of the metabolic model cannot be expected to capture genotypic and phenotypic adaptations beyond an initial adaptation. Here, the calculated growth reductions for the wild type and Δ*dosR* deletion mutant mimicked the experimental data up to days 60 and 12, respectively.

Figure 2 shows the predicted normalized oxygen uptake rates and ATP production rates for both wild type *M. tuberculosis* H37Rv and the corresponding Δ*dosR* mutant under normoxia and hypoxia 2 hours after switching to a condition of 0.2% oxygen. The oxygen uptake rates in Figure 2A were normalized by each strain's biomass production rate, for the different conditions, using the results in Supplemental Table S1 calculated as described in the Materials and Methods Section. While the normoxic conditions between the two strains were equivalent by construction, the hypoxic predictions between the two strains were quite different. The predicted wild type hypoxic oxygen uptake rate was substantially lower than the corresponding normoxic prediction, indicating that the wild type strain had the ability to substantially decrease its oxygen demand. At the same time, we only predicted a modest hypoxia-induced decrease in ATP production, suggestive of this strain's ability to maintain its energy production under low-oxygen stress. These predictions are qualitatively supported by experimentally observed lower ATP concentrations [16] for the wild type strain under hypoxia compared to normoxia. The hypoxic predictions for the Δ*dosR* strain differed substantially from the wild type predictions, suggesting that the deletion mutant was not able to modulate its metabolism to adapt to hypoxic conditions. In particular, the wild type normalized oxygen uptake rate was considerably lower than in the Δ*dosR* strain under hypoxic conditions, indicating that the deletion mutant was less able to adapt to the low-oxygen stress than the wild type per unit biomass. Because the deletion mutant is not able to grow efficiently under hypoxia, its overall ATP production rate is relatively lower than that for the wild type (Figure 2B). The predicted lower hypoxic ATP level and slower oxygen depletion in the Δ*dosR* mutant compared to the wild type have also been observed experimentally [49].

The inability of the Δ*dosR* strain to adapt to the low-oxygen environment is reflected in the difference in growth characteristics between the wild type and deletion mutant strains [49]. To test whether the strain- and condition-specific metabolic networks contain this growth information, we created corresponding *in silico* cellular growth predictions using an exponential growth model. We parameterized this model based on calculated growth rates and estimated lysis rates determined by fitting to the experimental cell concentrations of the wild type strain (see Materials and Methods for details). Figure 2C shows the experimentally determined *M. tuberculosis* cell concentrations during a 200-day growth period in which oxygen was depleted around day five, marking the onset of hypoxic growth [49]. This figure also shows the *in silico*-modeled cell concentrations of the two strains in the normoxic (days 0–5) and early hypoxic (days 5–60) stages of growth, and allows us, by inspection, to qualitatively estimate the time periods during which our model could capture the growth pattern of *M. tuberculosis*. The results for the wild type strain indicated that the model successfully reproduced the growth of this strain up to day 60. After this period, additional cellular reprogramming in response to extended hypoxia occurs [55], a response that is not dependent on *dosR* and represents further biological and metabolic adaptations not modeled here. The model results for the Δ*dosR* mutant provided relatively accurate prediction for the first 12 days. After this initial period, the model begins to break down, presumably due to additional biological and metabolic factors not modeled by the initial *dosR* gene reprogramming response.

To ascertain the robustness and specificity of our approach, we conducted *in silico* experiments to gauge the influence of fluctuations in the gene expression data and determine whether the metabolic predictions were specific to the expression data or the metabolic network *per se*.

**Figure 3:**
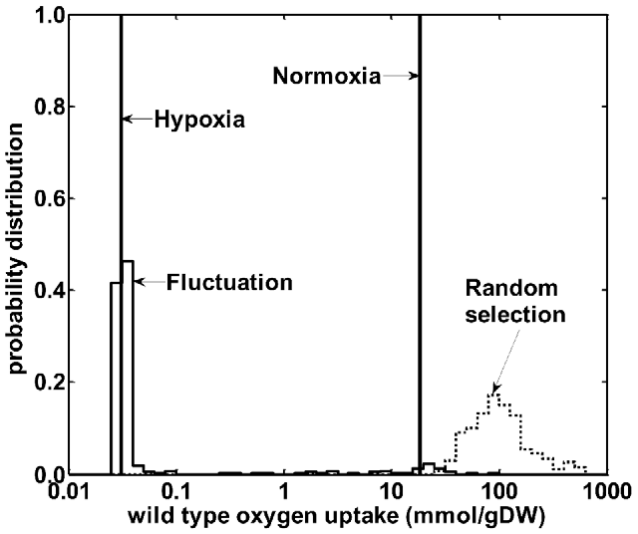
Variations of oxygen uptake of wild type *Mycobacterium tuberculosis* H37Rv are compatible with experimental fluctuations. The solid vertical bars indicate the wild type normoxic- and hypoxic-normalized oxygen uptake rates based on our integrated metabolic network model and correspond to the same values given in Figure 2A. We calculated the distribution of uptake values by sampling each expression value from a normalized distribution based on its mean and standard deviation and calculating the resultant uptake rates. This distribution, labeled “Fluctuations” in the graph, captured the experimental gene expression variability and was centered on the value derived from using the mean expression values. In contrast, the distribution labeled “Random selection” was derived from randomizing all gene expression data and was far away from either the normoxic or hypoxic conditions. This confirmed that the gene expression data carried sufficient information to guide the metabolic network model to describe the organism's adaptations under hypoxia. The units are mmol per gram dry weight of *Mycobacterium tuberculosis*.

To address fluctuations in the data, we created simulated gene expression data sets where all differential gene expression values were sampled from their corresponding normal distribution defined by their observed means and standard deviations [25]. For each data set, we calculated its hypoxic oxygen uptake rate, allowing us to re-construct a probability distribution of the hypoxic oxygen uptake rates for the wild type strain that is compatible with the given fluctuations of the experimental gene expression data. Figure 3 shows that 98% of the predicted oxygen uptake rates were centered on the hypoxic rate predicted using the mean experimental expression data, indicating that our predicted oxygen uptake was robust to fluctuations in gene expression measurements. Conversely, when we distributed the expression data randomly across genes in the metabolic network, the distribution of uptake rates was far from the originally predicted hypoxic value. This indicated that the predicted decrease in oxygen uptake stemmed directly from the specific changes in the gene expression data and was not an arbitrary result associated with random fluctuations in the metabolic network itself. Together, these results highlighted the strengths and limitations of using a model-based interpretation of metabolic adaptations as captured by differential gene expression data. The model correctly predicted the overall growth phenotype associated with the changed gene expression program, but if the gene expression program was subject to further changes, the model could not capture this without additional expression data.

### Changes in biomass composition of *M. tuberculosis* H37Rv under hypoxia

**Figure 4:**
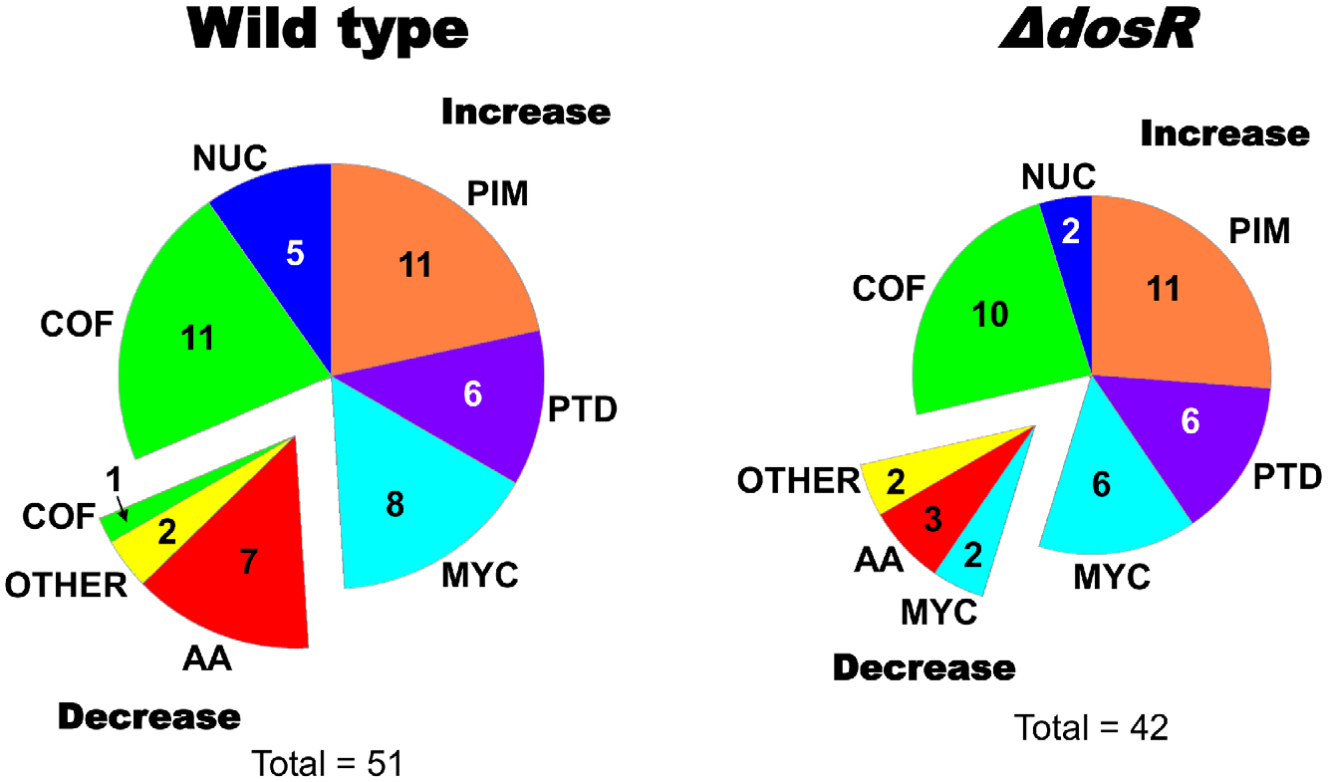
Classification of biomass metabolites predicted to change under hypoxia. Our metabolic network model predicted different adjustments in the biomass composition of wild type *Mycobacterium tuberculosis* and the Δ*dosR* deletion mutant under hypoxic stress. We classified these metabolites into the following categories: amino acids (AA), cofactors (COF), mycolates and related derivatives (MYC), nucleotides (NUC), phosphatidyl-myo-inositol mannosides (PIM), precursors of peptidoglycan (PTD), and other (OTHER). The pie charts indicate the numbers of metabolites that changed in each category. In total, the wild type was associated with 51 changes and the Δ*dosR* deletion mutant with 42 changes. The top-right portion in each chart represents the metabolites that were predicted to increase under hypoxia, while the bottom-left portion represents those predicted to decrease. Supplemental Table S2 provides detailed information for all predicted biomass composition changes.

Through the model interpretation of altered gene expression via the metabolic network, we predicted hypoxia-induced changes in biomass composition in both wild type *M. tuberculosis* H37Rv and the Δ*dosR* mutant. The prediction qualitatively indicated whether hypoxia induced an increase, decrease, or no change in each metabolite's biomass composition. Figure 4 shows the number of biomass metabolites in different biochemical categories predicted to increase and decrease due to hypoxia (Supplemental Table S2 provides the detailed list). For example, the figure indicates that, in the wild type strain, the biomass composition for five nucleotides (labeled as NUC in Figure 4) was predicted to increase under hypoxia while that for seven amino acids (AA in Figure 4) was predicted to decrease. Nearly half of the wild type predictions were associated with increased biomass composition of metabolites related to cell-wall components, such as mycolates (MYC), phosphatidyl-myo-inositol mannosides (PIM in Figure 4), and peptidoglycans (PTD in Figure 4) [56], [57]. These results were compatible with the experimentally observed thickening of the cell walls of mycobacteria during entry into hypoxia-induced dormancy [50], [51]. Other predictions for the wild type strain included increased nucleotide and decreased amino acid biomass composition, observations that are currently unsupported but should be the subject of future studies. When changes in biomass composition occurred for the Δ*dosR* mutant, they were similar to the wild type strain. However, the number of metabolites that were predicted to change for Δ*dosR* was slightly smaller than that for the wild type one (42 vs. 51), and the biomass compositions of two MYC-related metabolites that increased under hypoxia in the wild type strain actually decreased in Δ*dosR*. These results implied that the *dosR* gene played a role in the modulation of biomass composition, but not as a sole regulator of biomass accumulation. To further test the ability of our approach to alter biomass composition, we qualitatively predicted the biomass changes of *Mycobacterium bovis* upon transfer from a fast chemostat growth condition [36] to a slow growth condition [58] (see detailed results in Supplemental Text S2).

### Flux changes through central carbon metabolism of *M. tuberculosis* H37Rv under hypoxia

**Figure 5:**
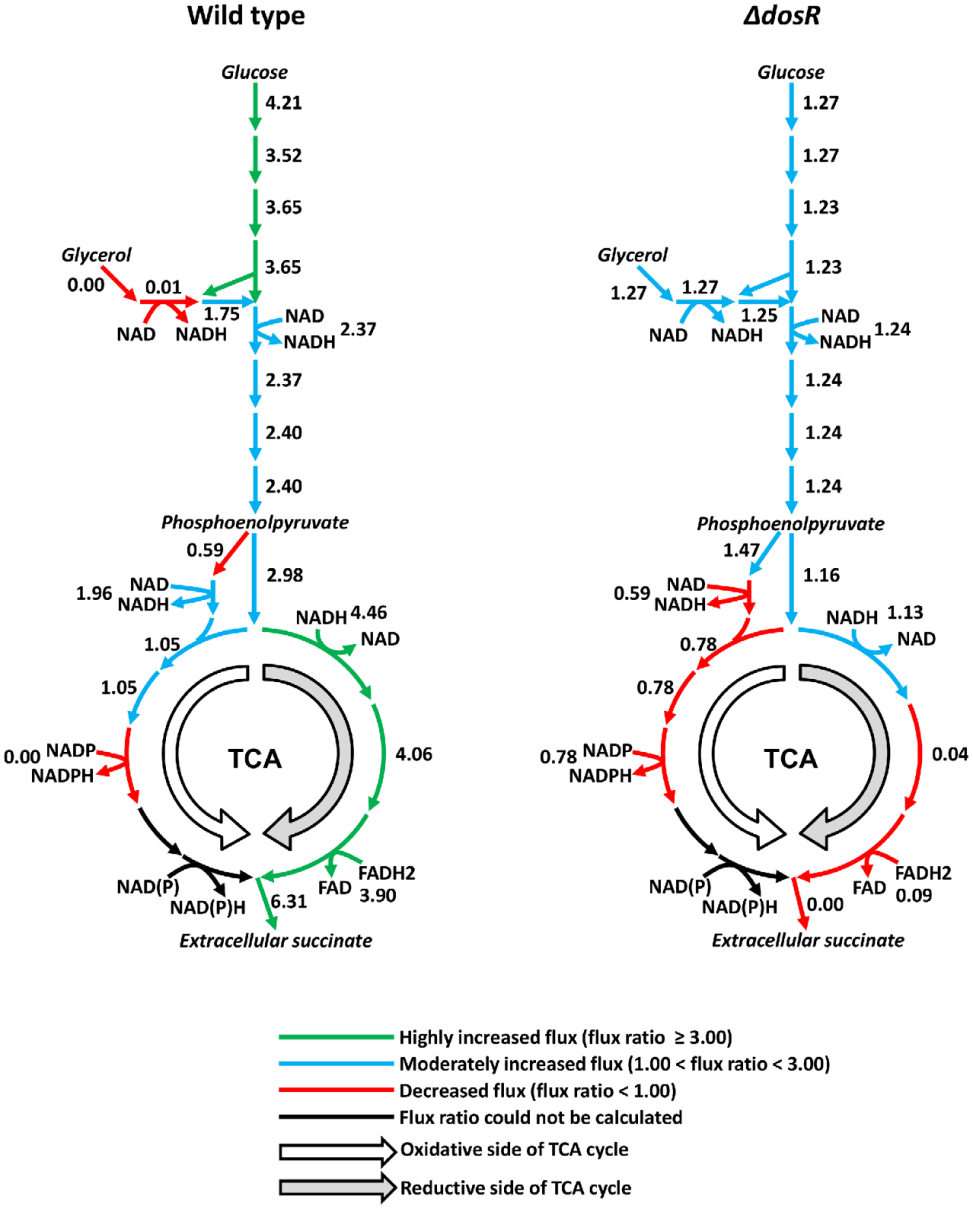
Predictions of hypoxia-induced changes in fluxes through central carbon metabolism. The left panel shows the flux ratios, i.e., the ratios of reaction fluxes under hypoxia to those under normoxia, of wild type *Mycobacterium tuberculosis* H37Rv and the right panel shows those of the Δ*dosR* deletion mutant. If the normoxic flux of a reaction was close to zero, we did not calculate the flux ratio for this reaction due to the numerical uncertainty associated with creating the corresponding ratio. The results indicated that the wild type strain activated glucose processing pathways and the predominant reaction flow was on the reductive side of the tricarboxylic acid (TCA) cycle. Conversely, the Δ*dosR* deletion mutant was not able to cope under hypoxic conditions as evident by an overall reduced activity in the TCA cycle. NAD, nicotinamide adenine dinucleotide; NADP, nicotinamide adenine dinucleotide phosphate; FAD, flavin adenine dinucleotide. NADH, NADPH, and FADH2 are the reduced forms of NAD, NADP, and FAD, respectively. NAD(P), NAD or NADP; NAD(P)H, NADH or NADPH.

Given that the metabolic network model provides detailed information of all metabolic fluxes, we examined the resulting hypoxia-induced changes associated with the carbon central metabolism of wild type *M. tuberculosis* H37Rv and its Δ*dosR* deletion mutant in more detail. Figure 5 shows that, in the wild type, the metabolite flux through the pathway associated with glucose utilization increased substantially while that through the glycerol utilization pathway decreased. Concomitantly, the flux through the reductive side of the TCA cycle increased considerably while that through the oxidative side only increased moderately. Conversely, the results for the Δ*dosR* strain indicated only a slight overall decrease in these fluxes. Thus, as captured by our integrated metabolic network model, without the *dosR* gene the organism fails to adapt its metabolism to cope with low-oxygen stress. Boshoff and coworkers recently confirmed the importance of fermentation in latent hypoxic *M. tuberculosis* by analyzing metabolite isotopes in the central carbon metabolism to identify the usage of the reductive TCA cycle under anaerobic conditions [16].

The altered flux distribution in the TCA cycle was also accompanied by altered extracellular secretion rates. In particular, the increased flux associated with succinate production at the bottom of the TCA cycle in Figure 5 produced an excess of succinate, which was secreted. We calculated that the hypoxic succinate secretion and accompanying H^+^ efflux was six times larger than the normoxic value of 9.1 millimoles per gram of dry weight of the organism (mmol/gDW). This was in qualitative agreement with experimentally observed succinate accumulation and acidification in the medium in which *M. tuberculosis* H37Rv is cultured under hypoxia [16].

**Figure 6:**
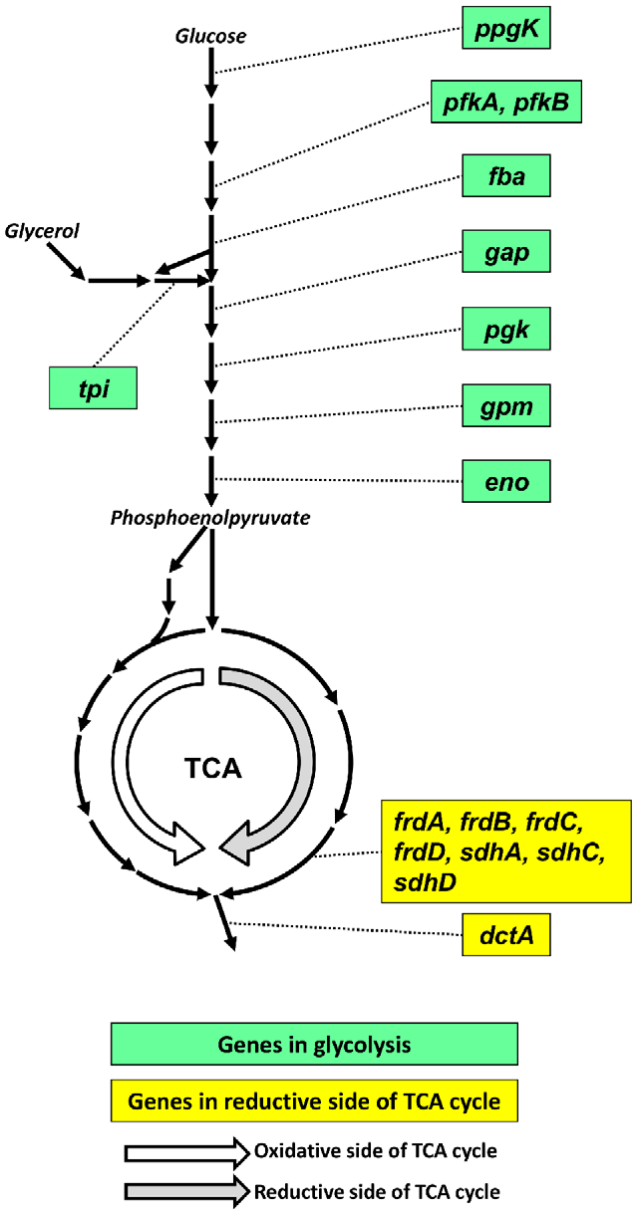
Genes predicted to be essential for *Mycobacterium tuberculosis* H37Rv to adapt to hypoxia. Shown are the genes predicted to be nonessential under normoxia but essential under hypoxia for the wild type strain. Given the metabolic state shown in Figure 5, the genes predicted to be essential for hypoxic adaptation were mostly located in the glucose/glycerol processing pathways and on the reductive side of the tricarboxylic acid (TCA) cycle. dctA, Na^+^/H^+^-dicarboxylate symporter; eno, enolase; fba, fructose-bisphosphate aldolase; frdA, frdB, frdC, frdD, fumarate reductase; gap, glyceraldehyde-3-phosphate dehydrogenase; gpm, phosphoglycerate mutase; pfKA, pfkB, phosphofructokinase; pgk, phosphoglycerate kinase; ppgK, polyphosphate glucokinase; sdhA, sdhC, sdhD, succinate dehydrogenase; tpi, triosephosphate isomerase.

We further characterized the altered metabolic state of the wild type strain associated with hypoxia by calculating which metabolic genes were essential for adaptation to hypoxic conditions. We defined these genes as those predicted to be nonessential under normoxia, i.e., removing them from the metabolic network did not prevent the organism from accumulating biomass, but became essential under hypoxia. Figure 6 shows that these genes were either in the glycolysis pathway or in the reductive side of TCA cycle, confirming that the two pathways were required for the hypoxic survival of *M. tuberculosis*. We further noted that due to the increased secretion of succinate mentioned above, the *dctA* gene that encodes for the succinate transporter was also predicted to become essential under hypoxia. This suggests that disruption of these pathways could prevent hypoxic adaptation and render the pathogen more susceptible to alternative antibiotic treatments.

Many CO_2_-fixating microbes utilize the reductive TCA cycle [59], but for hypoxic *M. tuberculosis* grown in glucose-supplemented Middlebrook 7H9 [25] or Dubos [16] media, the primary reason for utilizing glucose under reductive conditions is to maintain redox balance under low-oxygen availability. Under normoxia, *M. tuberculosis* oxidizes glucose and glycerol to carbon dioxide via glycolysis and the TCA cycle. At the same time, the oxidized forms of the redox intermediaries are converted to reduced forms in order to maintain chemical balance. Thus, the cell converts nicotinamide adenine dinucleotide (NAD), nicotinamide adenine dinucleotide phosphate (NADP), and flavin adenine dinucleotide (FAD) to the reduced forms of NADH, NADPH, and FADH2, respectively. Under normoxia, the cell maintains this balance by utilizing the constant supply of oxygen from the environment. Under hypoxia, to maintain balance of the redox intermediaries *M. tuberculosis* must decrease the reduction of NAD, NADP, or FAD and preferentially select pathways that convert the reduced forms back to the oxidized forms. Thus, in our model, hypoxic *M. tuberculosis* preferred glucose utilization because it reduces less NAD to NADH as compared to glycerol utilization and, as shown in Figure 5, and increased the flux through the reductive TCA cycle, as this pathway converts NADH and FADH2 back to NAD and FAD, respectively.

## Discussion

We developed a novel approach to interpret changes in gene expressions in terms of an altered metabolic program. Given a known reference state, we used changes in gene expression associated with a new state to construct a corresponding condition-specific metabolic state. This state captured the metabolic adaptations that the organism executed through an altered gene expression program. We modeled this adaptation via a metabolic network that characterizes nutrient uptake adjustments, alterations in preference of metabolic pathways, and changes in biomass composition. We implemented this approach to model the immediate change from aerobic to anaerobic conditions for *M. tuberculosis* and calculate its metabolic adaptations based only on differential gene expression data. Importantly, we derived our condition-specific metabolic states from gene expression data, which are widely available, and not protein abundances, which are rarely available.

### Integrating gene expression data and metabolic networks

There are a number of related methods that use gene expression data to modify the flow of metabolites in a metabolic network. These methods differ in their implementation of how transcription levels of different genes are connected to the reaction fluxes associated with the corresponding translated protein enzymes catalyzing the reactions. All methods deal differently with the general lack of a perfect correlation between transcriptional levels and protein concentrations and, hence, the lack of a direct one-to-one correspondence between expression values and reaction fluxes [60]. For example, in the Gene Inactivity Moderated by Metabolism and Expression (GIMME) procedure developed by Becker and Palsson [43], reactions that are associated with transcription levels lower than a fixed threshold are blocked. The method developed by Shlomi et al. [44] extended this approach by additionally forcing fluxes through reactions associated with high transcriptional levels. To avoid the determination of these somewhat arbitrary thresholds, the E-flux method introduced by Colijn et al. [37] uses transcriptional levels as the upper limits for the corresponding reaction fluxes. This method establishes such upper limits by using the absolute gene expression data to compute the relative changes of genes within the same treatment condition. In the Metabolic Adjustment by Differential Expression (MADE) method developed by Jensen and Papin [45], reaction fluxes are completely removed or unlimitedly allowed based on the corresponding relative gene expression levels between two conditions. The drawback of such a binary on/off approach is the lack of the ability to directly capture a gradual flux increase or decrease from the transcriptional data.

### Condition-specific models based on reference and treatment conditions

Our approach combined different aspects of the above methods by using the concepts of a reference and a treatment condition. For a treatment condition, our assumption was that an existing metabolic network could generate a set of reference fluxes characteristic of the reference condition and that the mRNA transcription data was reflective of the differential gene expression between the reference and treatment conditions. We used the relative expression changes to introduce soft constraints and limits on the relative flux changes, avoiding the introduction of arbitrary thresholds at the price of a more complex optimization problem. Regardless of whether an enzyme functions in either parallel or serial reaction paths in the metabolic network, we captured the notion that if there was a significant change in the expression level of a metabolic gene, it was very likely associated with an attempt to change the related reaction flux, although such a change is not required. In addition, we allowed the biomass composition to change in response to the treatment condition.

The advantage of this procedure was the general ability to account for all individual gene expression alterations and provide a detailed interpretation of the metabolic adjustments that capture gradual flux increases or decreases, without using any arbitrary threshold or assuming any correlation between absolute gene expression data and the upper limits of reaction fluxes across different genes under each condition. The disadvantages were in the formulation of a more complex optimization problem and the requirement of the availability of an existing metabolic network for the reference condition and the corresponding differential gene expression data between this reference condition and the treatment condition of interest.

### Strengths and limitations of using relative changes in gene expression levels

In our approach, we made use of differential gene expression data of the changes in the transcriptional program between a well-defined reference condition and a perturbed state. Because both mRNAs and proteins are under control of several different, but possibly correlated, processes, such as transcriptional and post-transcriptional control, degradation, ribosomal capacity, availability of the appropriate metabolites, and energy levels, the relative mRNA level of a gene is not necessarily directly proportional to the concentration of its corresponding protein [61]. However, relative changes in mRNA levels have been shown to be correlated to protein abundance in several studies of *Saccharomyces cerevisiae* (yeast) and prokaryotic bacteria. In yeast, the observed correlations between changes in mRNA level and protein abundance ranged from modest correlations between 0.2 and 0.5 [62]–[64] to a high of 0.7 [65]–[67]. Furthermore, these studies highlight the dependence of these values on the states of the studied organism, e.g., *S. cerevisiae* growing under steady state conditions shows a higher correlation between mRNA levels and protein abundance than under transient conditions [66]. In a study of *Escherichia coli* with a mutation in the *pgi* gene, transcription levels and corresponding protein abundance of the central metabolism genes changed in a correlated manner, with a coefficient of 0.81 [67]. Importantly, even in the studies that found weak correlations [62]–[64], the most strongly differentially expressed genes frequently displayed changes in mRNA level and protein abundance in the same direction. This has been verified in recent studies exploring transcriptomic and proteomic differences in both eukaryotic and prokaryotic single-celled organisms, with 88% [68] and 97% [69] of the genes with significantly altered transcription levels displaying changes in protein abundance in the same direction for *S. cerevisiae* and *Haemophilus influenzae*, respectively.

Thus, a formulation that uses a relative change correlation from one steady state to another could provide a practical approach under certain circumstances without requiring full knowledge of all possible regulatory mechanisms that govern the relationship between mRNA level and protein abundance. Our approach was based on the assumption that if the transcriptional mRNA level for a gene changes from a reference *ref* to an altered condition *new*, the limit of the corresponding normalized reaction flux catalyzed by the corresponding protein was bound by the ratio of [protein]*_new_*/[protein]*_ref_*. We approximated this ratio by [mRNA]*_new_*/[mRNA]*_ref_*+*L*, where the mRNA levels are taken from microarray experiments and *L* indicates a slack variable that allows for possible violation of the assumption. The method further assumes that the reference state is associated with a functioning metabolic network description that allows for steady state flux through its metabolite reactions and biomass accumulation. In addition, we assumed that the new state captured by the altered gene expression levels could also be described by a steady state approximation. This makes our model suitable for interpreting gene expression data that describes a transition from one stable condition to another one. In the case studied here, the immediate response of *M. tuberculosis* H37Rv to hypoxia, the model adaptations appear to be reliable for at least seven days after the insult. Longer-term adaptations that are dependent upon different gene expression programs, e.g., as in the extended hypoxic response, would have to be modeled by data directly associated with that state.

Metabolic adaptations associated with changing from an *in vitro* environment, where careful characterization of metabolism is possible, to an *in vivo* environment, where experimental data on metabolism would be more difficult to obtain, could be modeled based solely on differential gene expression measurements. Conversely, the metabolic model interpretation would not be suitable for creating tissue- or cell-specific metabolic networks based on the current overall human metabolic network reconstruction [70] because these use absolute rather than relative enzyme concentrations. However, if a cell-specific metabolic network exists that is amenable to constraint-based modeling, such as flux balance analysis (FBA), a change in the gene expression program of these cells due to some perturbation would be an excellent candidate for applying our methodology.

### Hypoxic adaptations of *M. tuberculosis*

The case study of modeling the immediate metabolic adaptation of *M. tuberculosis* H37Rv to hypoxia based on an existing metabolic network for normoxic conditions and relative gene expression changes under hypoxic conditions highlighted the type and amount of information that could be extracted from our modeling approach. Phenotypic effects of the hypoxia-induced gene expression program due to low-oxygen stress included adjusting the metabolism to a lower rate of oxygen uptake, lowering ATP utilization, altering biomass composition, increasing cell wall production, engaging the glucose-processing glycolysis pathway, and accommodating anaerobic respiration by using the reductive side of the TCA cycle. The *dosR* gene controlled this gene program and the gene expression profile of the deletion mutant Δ*dosR* revealed that it did not accommodate these metabolic adaptations. The mutant was less fit and displayed a substantially reduced growth rate under these conditions compared to the wild type strain. These predictions are supported by observations from previous experimental studies [49]–[51] and the recent confirmation of the importance of the reductive branch of the TCA cycle for latent tuberculosis [16]. The hypotheses regarding the altered importance of different enzymes under hypoxic conditions can be tested with gene knockout studies and, if validated, these enzymes may serve as novel drug target candidates for eliminating latent tuberculosis. In addition to the *in vitro*-based work presented here, our approach is ideally suited to elucidate the metabolic responses of *M. tuberculosis* to other stressors, such as nitric oxide [19] and carbon monoxide [20], as well as metabolic adaptations to animal-model-specific microenvironments [22].

## Materials and Methods

### *M. tuberculosis* HR37v metabolic network and gene expression data

We used the *in vitro iNJ*661m metabolic network of *M. tuberculosis* H37Rv [42], an enhanced version of the original *iNJ*661 network [35], as the reference network to describe cellular metabolism under normoxic growth conditions. The original *iNJ*661 model was augmented with reactions and metabolites involved in biotin synthesis, fumarate and succinate synthesis, and the methylcitrate cycle and minor changes to the biomass function were made. The *iNJ*661m network contains 663 genes, 838 metabolites, and 1,049 reactions and correctly predicts growth rates of normoxic H37Rv in different media. We used microarray data measured in triplicate from Park et al. [25] as the source for differential gene expression associated with the transcriptomic alteration two hours after the transfer from normoxic air to hypoxic nitrogen gas with 0.2% oxygen (1.5 mm Hg) for both wild type *M. tuberculosis* H37Rv and the Δ*dosR* deletion mutant [25]. Out of the 501 genes that showed a more than 1.8-fold change, 96 appeared in the metabolic network, 16 of which were down-regulated and 80 were up-regulated.

### Integrating a reference metabolic network and differential gene expression data

Figure 1 shows the overall scheme for integrating a given metabolic network compatible with a reference condition and a set of differential gene expression data describing mRNA transcription changes going from the reference state to the new state. Our method depended on developing a set of constraints (Steps I–IV) that take into account the known metabolic reference conditions and possible alterations in metabolite flow through any given reaction associated with an expression change to produce a metabolic representation of these constraints (Step V).

#### Step I

In this step, we first performed a flux variability analysis (FVA) [71], [72] to calculate the minimum and maximum fluxes through each reaction *i* under an FBA-predicted optimal biomass production rate. Further, we solved an optimization problem to obtain a set of reference fluxes *x_i,ref_* for each reaction *i* normalized by the optimal biomass production rate (see Supplemental Text S3 for details). This reference flux distribution satisfied all necessary constraints and was close to the means of the normalized minimum and maximum fluxes (i.e., the minimum and maximum fluxes divided by the reference optimal biomass production rate [36]). Therefore, this reference state is representative of the reference condition, in our case *M. tuberculosis* growth under normoxic conditions, and should be a good starting point for determining alterations in fluxes in the perturbed hypoxic state based on altered gene expressions. The renormalization of the fluxes with the optimal biomass growth rate was necessary to transform the optimization from a non-linear to a linear problem and improve numerical stability. In applications of the method in which experimentally measured reference fluxes are available, these could instead be used as reference fluxes.

#### Step II

We associated gene expression ratios with reaction ratios according to the gene-protein-reaction relations in the metabolic network. If only one gene was associated with a metabolic reaction *i*, the expression ratio of this gene was assigned to the reaction ratio *R_i_*. If several genes were jointly required for a reaction *i* to take place, the reaction ratio *R_i_* was assigned to the geometric mean of the expression ratios of the genes. This formulation captured the condition that all reaction ratios were required. If any one of several genes was sufficient for a reaction *i* to occur, the reaction ratio *R_i_* was assigned to the arithmetic mean of the expression ratios of the genes. This formulation captured the condition that, at a minimum, any one reaction ratio was required. If a gene (or genes) was associated with several reactions, we used the above rules to construct a reaction ratio and assign this ratio to the overall normalized flux of these reactions.

#### Step III

Given the values for *x_i,ref_* and *R_i_* obtained above, we developed a set of constraints to incorporate the effects of altered gene expression on the metabolic network (uptake rates *U*, reaction fluxes *x*, and biomass composition *c*). We summarized these constraints in Equations 1–6 (a complete technical description is provided in Supplemental Text S4) and describe their most important features below:
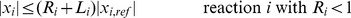
(1)

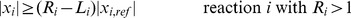
(2)

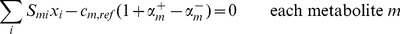
(3)

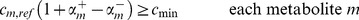
(4)

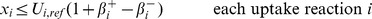
(5)

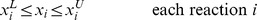
(6)


Constraints 1 and 2 describe how gene expression changes could affect reaction fluxes in our model, with the special cases of reversible and irreversible reaction fluxes treated more fully in Supplemental Text S4. If the gene(s) related to a reaction *i* was down-regulated (*R_i_*<1), we attempted to decrease the absolute value for *x_i_*, the normalized flux through the reaction, to the level of *R_i_*|*x_i,ref_*| (the converse holds for up-regulated genes). To allow for possible violations of the corresponding constraints, we introduced non-negative slack variables *L_i_* to modulate the flux level constraints.

Constraints 3 and 4 account for possible modifications in the biomass objective function and determine the new biomass composition *c_m,new_*. Constraint 3 represents the mass balance of each metabolite, where *S_mi_* denotes the stoichiometric coefficient for metabolite *m* in reaction *i*, *c_m,ref_* represents the original coefficient of this metabolite in the biomass objective function, and the non-negative variables 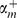 and 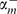 indicate the possible increase and decrease, respectively, of the coefficient. The biomass modification was further constrained to a lower limit *c_min_* for each metabolite (Equation 4). The biomass coefficient under the new condition *c_m,new_* was equal to *c_m,ref_* (1+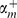
−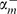).

Constraints 5 and 6 determine the upper limit of the normalized flux of each uptake and intracellular reaction flux. *U_i,ref_* denotes the upper limit in the reference condition, which is equal to the original upper limit in the original metabolic network divided by the reference optimal biomass production rate, and the non-negative variables 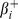 and 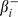 indicate the possible increase and decrease in the normalized limit. The new upper limit *U_i,new_* was determined as 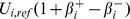. The normalized flux through a non-uptake reaction, *x_i_*, was constrained between its lower (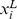) and upper bounds (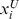) determined from corresponding original lower and upper bounds in the original reference metabolic network divided by the reference optimal biomass production rate.

#### Step IV

Subject to the developed constraints, we minimized the sum of the slack variables (Σ*L_i_*) and then minimized the modifications in the biomass objective function (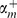 and 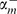) and in the upper limits of the metabolite uptakes (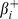 and 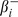). Finally, we obtained the minimum and maximum normalized flux through each reaction. Additional technical details are provided as Supplemental Text S5.

#### Step V

We obtained a distribution of the normalized fluxes under the new condition, *x_i,new_*, by solving an optimization problem similar to the problem S5–S8 in Supplemental Text S3. In particular, we minimized the sum of the absolute differences between *x_i,new_* and means of the minimum and maximum fluxes calculated in Step IV, subject to all necessary constraints (Constraints S11–S20 with the determined values for minimum Σ*L_i_*, 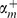, 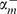, 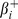, and 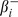 and the limiting oxygen uptake reaction flux, see Supplemental Text S4 and S5 for details). If the corresponding reference flux *x_i,ref_* was not equal to zero, we constructed a flux ratio with respect to the new condition as the absolute value of the ratio of *x_i,new_* to *x_i,ref_*.

Model construction, data processing, and simulations were carried out in MATLAB (2011b, MathWorks, Natick, MA) using the COBRA toolbox [73]. The metabolic models (in MATLAB format) and parameter sets for simulating both wild type and the Δ*dosR* deletion mutant are provided as Supplemental Protocol S1.

### Analysis of fluctuations and randomization of gene expression data

We performed 500 Monte Carlo simulations to calculate the distribution of normalized oxygen uptake rates of the wild type strain based on experimentally determined gene expression fluctuations. In each simulation, we randomly generated an expression value for each gene based on its assumed normal distribution with mean and standard deviation corresponding to the experimental wild type values [25], and used Steps I–V to calculate the normalized oxygen uptake.

We also performed another set of 500 simulations to calculate the oxygen uptake rates for randomized gene expression data sets. In these simulations, we assigned an expression value for each gene by randomly selecting a value from the experimental data set [25] and used Steps I–V to calculate the normalized oxygen uptake rate for each randomized gene set.

### Calculation of cell concentrations during normoxic and hypoxic growth

We calculated the cell concentrations of wild type *M. tuberculosis* H37Rv and the Δ*dosR* deletion mutant under normoxic (days 0–5) and early hypoxic (days 5–60) conditions to compare the model predictions with experimentally determined growth characteristics [49]. Using the initial cell concentrations [49], we solved the following ordinary differential equation:
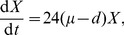
(7)where *X* indicates the cell concentration of *M. tuberculosis*, *t* denotes time in days, and *μ* and *d* represent biomass production rate and lysis rate, respectively, in units of h^−1^. The value for *μ* differed between the strains of *M. tuberculosis* (wild type and Δ*dosR*) under the two growth conditions (normoxic and hypoxic), while we assigned *d* one uniform value and assumed that this value was the same for both strains. The parameters of this equation were determined from matching the calculated growth of the wild type strain to the experimental values. We first performed an FBA of the *iNJ*661m network [42] to calculate the wild type *wt* strain's normoxic *n* biomass production rate *μ_wt,n_* and then determined the value of *d* by reproducing the experimental normoxic cell concentrations. Given *d*, we further matched the calculated cell concentrations under hypoxia of the wild type to determine the hypoxic *h* biomass production rate *μ_wt,h_*. To estimate the growth rate of the Δ*dosR* mutant, we assumed an inverse proportionality between normalized oxygen uptake rate and biomass production rate. Thus, we set the normoxic biomass production rate *μ*_Δ*dosR,n*_ to be equal to that of the wild type strain *μ_wt,n_*, and obtained the hypoxic rate of the mutant *μ*_Δ*dosR,h*_ via the following equation:
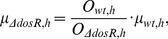
(8)where *O_wt,h_* and *O*_Δ*dosR,h*_ denote the calculated normalized oxygen uptakes of the wild type and Δ*dosR* strains under hypoxia, respectively. Supplemental Table S1 provides all condition- and strain-specific values for the biomass production rate *μ* and lysis rate *d*.

### Identification of metabolic genes essential for *M. tuberculosis* to adapt to hypoxia

We identified a metabolic gene as being essential for adaptation to hypoxia if we predicted that this gene was nonessential under normoxia but essential under hypoxia. To determine gene essentiality under normoxia, we performed an FBA to predict the biomass production rate for the wild type strain and for each individual metabolic gene deletion mutant. We modeled deletion mutants by removing all reaction(s) related to the deleted gene. If the ratio for the biomass production rate of a single-gene deletion mutant to wild type was greater than a threshold (0.10), we categorized the metabolic gene as nonessential under normoxia. Similarly, if the ratio for the biomass production rate calculated under hypoxia (as approximated in Equation 8) was less than the threshold (0.10), we categorized the corresponding gene as essential under hypoxia. All calculated ratios were either >0.25 or <0.01 and the choice of 0.10 was thus robust with respect to differentiating ratios close to zero from those significantly higher than zero.

## Supporting Information

Protocol S1 **MATLAB-formatted models of metabolism for** ***M. tuberculosis*** **HR37v and its Δ** ***dosR*** **deletion mutant.**
(ZIP)

Table S1 **Biomass production and cellular lysis rates for simulating growth of wild type** ***M. tuberculosis*** **H37Rv and the Δ** ***dosR*** **deletion mutant under normoxia and hypoxia.**
(PDF)

Table S2 **Predictions of the hypoxia-induced changes in biomass composition of wild type** ***M. tuberculosis*** **H37Rv and the Δ** ***dosR*** **deletion mutant.** Normoxic coefficients were set to be the same as the original coefficients of the corresponding metabolites in the biomass function of the *iNJ*661m metabolic network [42]. The hypoxic coefficients were predicted by integrating the metabolic network with gene expression data for the wild type strain (or the Δ*dosR* deletion mutant strain) of *Mycobacterium tuberculosis* H37Rv under hypoxia [25]. Changes in the coefficients indicated an altered biomass composition of *M. tuberculosis* H37Rv upon exposure to hypoxia. “Increase” (or “Decrease”) indicated that a predicted hypoxic coefficient was greater (or smaller) than the corresponding normoxic coefficient.
(PDF)

Text S1 **Predicting metabolic fluxes in yeast.**
(PDF)

Text S2 **Biomass change of** ***M. bovis*** **under slow growth conditions.**
(PDF)

Text S3 **Generation of a reference flux distribution in Step I.**
(PDF)

Text S4 **Additional implementation details for the constraints given in Step III.**
(PDF)

Text S5 **Detailed descriptions for solving the optimization problems outlined in Step IV.**
(PDF)
