# Supplementary material for: Modeling Phenotypic Metabolic Adaptations of Mycobacterium tuberculosis H37Rv under Hypoxia
Source: PLoS Comput Biol. 2012 Sep 13;8(9):e1002688. doi: 10.1371/journal.pcbi.1002688 (PMC3441462; doi:10.1371/journal.pcbi.1002688)
Supplement: Protocol S1 — MATLAB-formatted models of metabolism for M. tuberculosis HR37v and its Δ dosR deletion mutant. (ZIP) [file pcbi.1002688.s001.zip › README.pdf]

System requirements:

To run the attached MATLAB scripts, the software packages below have to be installed on the computer.

Software	Developer	Related website
MATLAB2010 or 2011	MathWorks, Inc.	http://www.mathworks.com/
The COnstraints Based Reconstruction and Analysis (COBRA) Toolbox	Systems Biology Research Group at University of California, San Diego	http://opencobra.sourceforge.net/openCOBRA/Welcome.html
GNU Linear Programming Kit (GLPK)	Free Software Foundation, Inc.	http://www.gnu.org/software/glpk/
MATLAB Interface for GLPK	Nicolo Giorgett	http://glpkmex.sourceforge.net/
libSBML	Sarah Keating, Akiya Jouraku, Frank Bergmann, Ben Bornstein, Michael Hucka, and others	http://sbml.org/Software/libSBML
SBMLToolbox	Keating, S. M., Bornstein, B. J., Finney, A., and Hucka M.	http://sbml.org/Software/SBMLToolbox

How to run the attached MATLAB scripts:

1) Install the above software packages by following the instructions in the provided links;
2) Unzip all files attached with this article to a work directory;
3) Open MATLAB;

4) Change “Current Folder” in the MATLAB environment to the directory where COBRA Toolbox was installed;
5) In the command window of the MATLAB environment, run the command “initCobraToolbox”;
6) Change “Current Folder” in the MATLAB environment to the work directory where the files attached with this article were placed;
7) In the command window of the MATLAB environment, run the command “genelistdosRWT_relative061012” to calculate the metabolic response of wild type *Mycobacterium tuberculosis* to hypoxia;
8) In the command window of the MATLAB environment, run the command “genelistdosRKO_relative061012” to calculate the *ΔdosR* gene deletion mutant of *Mycobacterium tuberculosis* to hypoxia.

Disclaimer

The supplemented script codes and other related files are provided “as is” and “with all faults.” We, the authors of the related manuscript, make no representations or warranties of any kind concerning the safety, suitability, lack of viruses, inaccuracies, typographical errors, or other harmful components of the supplemented files. There are inherent dangers in the use of any software, and you are solely responsible for determining whether the supplemented files are compatible with your equipment and other software installed on your equipment. You are also solely responsible for the protection of your equipment and backup of your data, and we will not be liable for any damages you may suffer in connection with using, modifying, or distributing the supplemented files.
