# Supplementary material for: Modeling Phenotypic Metabolic Adaptations of Mycobacterium tuberculosis H37Rv under Hypoxia
Source: PLoS Comput Biol. 2012 Sep 13;8(9):e1002688. doi: 10.1371/journal.pcbi.1002688 (PMC3441462; doi:10.1371/journal.pcbi.1002688)
Supplement: Table S1 — Biomass production and cellular lysis rates for simulating growth of wild type M. tuberculosis H37Rv and the Δ dosR deletion mutant under normoxia and hypoxia. (PDF) [file pcbi.1002688.s002.pdf]

Supplemental Table S1: Biomass production and cellular lysis rates for simulating growth of wild type *Mycobacterium tuberculosis* H37Rv and the Δ*dosR* deletion mutant under normoxia and hypoxia.

	Biomass production rate (h^{-1})		Lysis rate (h^{-1})
	Wild type	Δ*dosR*	
Normoxia (≤ 4 days)	5.2×10^{-2}	5.2×10^{-2}	1.2×10^{-2}
Hypoxia (≥ 6 days)	1.1×10^{-2}	1.4×10^{-5}	1.2×10^{-2}
