# Supplementary material for: Modeling Phenotypic Metabolic Adaptations of Mycobacterium tuberculosis H37Rv under Hypoxia
Source: PLoS Comput Biol. 2012 Sep 13;8(9):e1002688. doi: 10.1371/journal.pcbi.1002688 (PMC3441462; doi:10.1371/journal.pcbi.1002688)
Supplement: Table S2 — Predictions of the hypoxia-induced changes in biomass composition of wild type M. tuberculosis H37Rv and the Δ dosR deletion mutant. Normoxic coefficients were set to be the same as the original coefficients of the corresponding metabolites in the biomass function of the iNJ661m metabolic network [42]. The hypoxic coefficients were predicted by integrating the metabolic network with gene expression data for the wild type strain (or the ΔdosR deletion mutant strain) of Mycobacterium tuberculosis H37Rv under hypoxia [25]. Changes in the coefficients indicated an altered biomass composition of M. tuberculosis H37Rv upon exposure to hypoxia. “Increase” (or “Decrease”) indicated that a predicted hypoxic coefficient was greater (or smaller) than the corresponding normoxic coefficient. (PDF) [file pcbi.1002688.s003.pdf]

Supplemental Table S2: Predicted hypoxia-induced changes in biomass composition of wild type *Mycobacterium tuberculosis* H37Rv and the Δ*dosR* deletion mutant.

Normoxic coefficients were set to be the same as the original coefficients of the corresponding metabolites in the biomass function of the *iNJ*661m metabolic network [42]. The hypoxic coefficients were predicted by integrating the metabolic network with gene expression data for the wild type strain (or the Δ*dosR* deletion mutant strain) of *Mycobacterium tuberculosis* H37Rv under hypoxia [25]. Changes in the coefficients indicated an altered biomass composition of *M. tuberculosis* H37Rv upon exposure to hypoxia. "Increase" (or "Decrease") indicated that a predicted hypoxic coefficient was greater (or smaller) than the corresponding normoxic coefficient.

Index	Metabolite name	Normoxic coefficient in biomass	Wild type		Δ*dosR*		Note
			Hypoxic coefficient in biomass	Change in the coefficient	Hypoxic coefficient in biomass	Change in the coefficient	
1	Acyl phosphatidylinositol mannoside	1.7×10^{-3}	5.4×10^{-3}	Increase	6.6×10^{-3}	Increase	Phosphatidy linositol mannosides, cell-wall components [57]
2	Acyl phosphatidylinositol mannoside di-mannose	1.5×10^{-3}	4.7×10^{-3}	Increase	5.8×10^{-3}	Increase	
3	Acyl phosphatidylinositol mannoside tri-mannose	1.3×10^{-3}	4.3×10^{-3}	Increase	5.2×10^{-3}	Increase	
4	Acyl phosphatidylinositol mannoside tetra-mannose	1.2×10^{-3}	3.9×10^{-3}	Increase	4.7×10^{-3}	Increase	
5	Di-acyl phosphatidylinositol mannoside di-mannose	1.3×10^{-3}	4.1×10^{-3}	Increase	5.0×10^{-3}	Increase	
6	Phosphatidylinositol mannoside	2.1×10^{-3}	6.6×10^{-3}	Increase	8.1×10^{-3}	Increase	
7	Phosphatidylinositol mannoside di-mannose	1.8×10^{-3}	5.7×10^{-3}	Increase	7.0×10^{-3}	Increase	
8	Phosphatidylinositol mannoside tri-mannose	1.6×10^{-3}	5.0×10^{-3}	Increase	6.1×10^{-3}	Increase	
9	Phosphatidylinositol mannoside tetra-mannose	1.4×10^{-3}	4.5×10^{-3}	Increase	5.5×10^{-3}	Increase	
10	Phosphatidylinositol mannoside penta-mannose	1.3×10^{-3}	4.1×10^{-3}	Increase	4.9×10^{-3}	Increase	
11	Phosphatidylinositol mannoside hexa-mannose	1.2×10^{-3}	3.7×10^{-3}	Increase	4.5×10^{-3}	Increase	

12	Undecaprenyl-diphospho-N-acetylmuramoyl-N-acetylglucosamine-L-alanyl-D-glutamyl-L-lysyl-D-alanyl-D-alanine	1.0×10^{-3}	2.1×10^{-3}	Increase	2.4×10^{-3}	Increase	Precursors of peptido-glycan, a cell-wall component [56]
13	Undecaprenyl-diphospho-N-acetylmuramoyl-N-acetylglucosamine-L-alanyl-gamma-D-glutamyl-L-lysyl-D-alanyl-D-alanine	1.0×10^{-3}	2.1×10^{-3}	Increase	2.4×10^{-3}	Increase	
14	Undecaprenyl-diphospho-N-acetylmuramoyl-N-acetylglucosamine-L-ala-D-glu-meso-2-6-diaminopimeloyl-D-ala-D-ala	9.9×10^{-4}	2.1×10^{-3}	Increase	2.4×10^{-3}	Increase	
15	Undecaprenyl-diphospho-N-glycolylmuramoyl-N-acetylglucosamine-L-ala-D-glu-meso-2-6-diaminopimeloyl-D-ala-D-ala	9.8×10^{-4}	2.1×10^{-3}	Increase	2.3×10^{-3}	Increase	
16	Peptidoglycan subunit1	1.9×10^{-2}	4.0×10^{-2}	Increase	4.5×10^{-2}	Increase	
17	Peptidoglycan subunit2	1.9×10^{-2}	4.0×10^{-2}	Increase	4.5×10^{-2}	Increase	
18	Keto mycolate (2 cyclopropanated rings)	3.3×10^{-3}	8.2×10^{-3}	Increase	1.1×10^{-2}	Increase	Mycolates and related derivatives, cell-wall components [56]
19	Keto mycolate (1 cyclopropanated ring)	3.3×10^{-3}	8.1×10^{-3}	Increase	5.0×10^{-7}	Decrease	
20	Methoxy mycolate (1 cyclopropanated ring)	3.4×10^{-3}	8.3×10^{-3}	Increase	5.0×10^{-7}	Decrease	
21	Mycolate (2 cyclopropanated rings)	1.1×10^{-2}	2.7×10^{-2}	Increase	3.7×10^{-2}	Increase	
22	Tetramycolyl hexaarabinoside 1	1.1×10^{-3}	2.7×10^{-3}	Increase	3.7×10^{-3}	Increase	
23	Tetramycolyl hexaarabinoside 2	1.1×10^{-3}	2.7×10^{-3}	Increase	3.7×10^{-3}	Increase	
24	Tetramycolyl hexaarabinoside 3	1.1×10^{-3}	2.7×10^{-3}	Increase	3.7×10^{-3}	Increase	
25	Tetramycolyl hexaarabinoside 4	1.1×10^{-3}	2.7×10^{-3}	Increase	1.3×10^{-3}	Increase	

26	Adenosine monophosphate (AMP)	1.4×10^{-1}	1.8×10^{-1}	Increase	1.4×10^{-1}	No change	Nucleotides
27	Guanosine monophosphate (GMP)	2.4×10^{-1}	3.1×10^{-1}	Increase	3.2×10^{-1}	Increase	
28	Deoxyadenosine monophosphate (dAMP)	3.5×10^{-3}	6.1×10^{-3}	Increase	3.5×10^{-3}	No change	
29	Deoxyguanosine monophosphate (dGMP)	6.7×10^{-3}	8.5×10^{-3}	Increase	8.8×10^{-3}	Increase	
30	Deoxythymidine monophosphate (dTMP)	3.7×10^{-3}	6.4×10^{-3}	Increase	3.7×10^{-3}	No change	
31	L-Cysteine	2.2×10^{-2}	1.6×10^{-2}	Decrease	2.2×10^{-2}	No change	Amino acids
32	Glycine	3.4×10^{-1}	5.0×10^{-7}	Decrease	5.0×10^{-7}	Decrease	
33	L-Methionine	3.5×10^{-2}	2.5×10^{-2}	Decrease	3.5×10^{-2}	No change	
34	L-Phenylalanine	4.8×10^{-2}	2.2×10^{-2}	Decrease	2.0×10^{-2}	Decrease	
35	L-Serine	1.4×10^{-1}	1.0×10^{-1}	Decrease	1.4×10^{-1}	No change	
36	L-Tryptophan	2.0×10^{-2}	1.5×10^{-2}	Decrease	2.0×10^{-2}	No change	
37	L-Tyrosine	3.2×10^{-2}	1.4×10^{-2}	Decrease	1.3×10^{-2}	Decrease	
38	5-6-7-8-Tetrahydrofolate	1.0×10^{-6}	1.8×10^{-6}	Increase	2.3×10^{-6}	Increase	Cofactors
39	7-8-Dihydrofolate	1.0×10^{-6}	1.8×10^{-6}	Increase	2.3×10^{-6}	Increase	
40	Menaquinol-8	1.0×10^{-6}	1.9×10^{-6}	Increase	1.0×10^{-6}	No change	
41	Nicotinamide adenine dinucleotide (NAD)	1.0×10^{-6}	2.8×10^{-6}	Increase	3.1×10^{-6}	Increase	
42	Nicotinamide adenine dinucleotide phosphate (NADP)	1.0×10^{-6}	2.8×10^{-6}	Increase	3.1×10^{-6}	Increase	
43	Quinolinate	1.0×10^{-6}	2.8×10^{-6}	Increase	3.1×10^{-6}	Increase	
44	Protoheme	1.0×10^{-6}	2.5×10^{-6}	Increase	3.2×10^{-6}	Increase	
45	Thiamin	1.0×10^{-6}	1.9×10^{-6}	Increase	2.3×10^{-6}	Increase	
46	Hydroxymethylbilane	1.0×10^{-6}	1.9×10^{-6}	Increase	2.6×10^{-6}	Increase	
47	Flavin mononucleotide	1.0×10^{-6}	1.3×10^{-6}	Increase	1.3×10^{-6}	Increase	
48	Riboflavin	1.0×10^{-6}	1.3×10^{-6}	Increase	1.3×10^{-6}	Increase	
49	Pantetheine 4'-phosphate	1.0×10^{-6}	7.2×10^{-7}	Decrease	1.0×10^{-6}	No change	

50	Iron-III-chelated carboxymycobactin-T	1.5×10^{-3}	1.0×10^{-3}	Decrease	1.5×10^{-3}	No change	Other
51	Phenolic glycolipid	1.2×10^{-3}	1.2×10^{-3}	No change	6.6×10^{-4}	Decrease	
52	Phosphatidylethanolamine (dihexadecanoyl, n-C16:0)	4.1×10^{-3}	2.9×10^{-3}	Decrease	4.1×10^{-3}	No change	
53	Phenol phthiocerol dimycocerosate	2.6×10^{-4}	2.6×10^{-4}	No change	1.5×10^{-4}	Decrease	
