# Supplementary material for: Modeling Phenotypic Metabolic Adaptations of Mycobacterium tuberculosis H37Rv under Hypoxia
Source: PLoS Comput Biol. 2012 Sep 13;8(9):e1002688. doi: 10.1371/journal.pcbi.1002688 (PMC3441462; doi:10.1371/journal.pcbi.1002688)
Supplement: Text S1 — Predicting metabolic fluxes in yeast. (PDF) [file pcbi.1002688.s004.pdf]

Supplemental Text S1. Predicting fluxes in yeast.

As an initial validation, we used our approach to predict metabolic fluxes in yeast under different conditions, and compared the predictions with experimental measurements.

Wild type yeast grown on different carbon sources

Daran-Lapujade et al. studied the responses of yeast to different carbon sources, by measuring reaction fluxes in the central carbon metabolism and gene expression levels of yeast in four media, each of which was infused with glucose, maltose, ethanol, or acetate as the single carbon source [1]. By using our approach, we first generated a reference flux distribution representative of yeast metabolism in the glucose medium (see Supplemental Text S3 for the detailed method). Given these reference fluxes, we predicted a flux distribution for yeast in each of the other three media, through the integration of the yeast metabolic network presented by Moxley et al. [2] with the relative gene expression difference between yeast in the glucose medium and in each of the three media [1].

Supplemental Table S3 shows the comparison between the predicted and experimental oxygen uptake rates and carbon dioxide secretion rates of yeast for the four carbon sources, with an average relative difference of 9% between the predictions and experimental measurements. We compared the predicted metabolic fluxes in the central carbon metabolism with the corresponding experimental measurements (see Supplemental Figure S1). For yeast grown with glucose, maltose, ethanol, and acetate as the single carbon source, the predicted and experimentally measured fluxes exhibited good correlations (Pearson correlation coefficients

ranging from 0.61 to 0.79), illustrating the ability of our approach to predict metabolic fluxes.

Wild type yeast and the Δgcn4 deletion mutant under histidine starvation stress

Moxley et al. investigated the role of the transcription factor Gcn4p in gene regulation and metabolism in yeast under histidine starvation stress [2]. They measured gene expression of wild type yeast and its Δ*gcn4* deletion mutant in chemostat media infused with 3-amino-1,2,4-triazole, a histidine synthesis inhibitor, and determined the corresponding metabolic fluxes of the two strains by experimentally measuring distributions of ^{13}C isotope isomers of metabolites [2]. By using our approach, we integrated the same gene expression data and the yeast metabolic network presented by Moxley to predict how the deletion of the *gcn4* gene would change the metabolic fluxes, and compared our predictions with the experimentally determined fluxes [2]. Because yeast is under histidine starvation stress and, thus, is not at its optimal growth, we did not use the computational method given in Supplemental Text S3 to generate the reference fluxes, but instead set them to be equal to the available experimentally measured fluxes in wild type yeast [2].

In order to categorize a flux change as a significant change, we examined the distribution of flux changes to determine a threshold value. The distribution (data not shown) was peaked around zero but not normally distributed. After some trial and error, we selected the median of the absolute value of the experimentally measured flux differences as characteristic of the width of the distribution and used this value T as a threshold to characterize changes. Thus, a reaction flux was classified as increasing, decreasing, or exhibiting no change, when the difference between the corresponding Δ*gcn4* and wild type fluxes was greater than T, smaller than $-T$, or between $-T$

and *T*, respectively. We assigned *T* to be 1.3 in the arbitrary units used by Moxley et al. [2].

Supplemental Table S4 shows the number of reaction fluxes that increased, decreased, or exhibited no change when the *gcn4* gene was removed from yeast as determined experimentally or computationally. Fisher's exact test of the 3×3 contingency table gave a *p*-value of 1×10^{-5}, indicating that we can reject the null hypothesis that the association between the qualitative prediction of flux changes and the corresponding experimental observation was due to chance. We further evaluated whether the association between the predicted and experimentally measured flux changes were still significant under different values for *T*. Supplemental Figure S2 shows that the *p*-values from Fisher's exact test was smaller than 0.05 when the threshold *T* ranged from 0.2 to 100, suggesting that the *p*-value estimate was robust to changes in the threshold.

In addition, we also directly compared the predicted quantitative flux differences (Δ*gcn4* minus wild type) with the corresponding experimental data [2]. When we considered all reaction fluxes (90 fluxes), we obtained a modest correlation between the predicted and experimental flux differences (Pearson correlation coefficient = 0.52). However, when we only considered the set of fluxes selected by Moxley et al. [2] as being of high-confidence (24 fluxes), we obtained a correlation coefficient of 0.79 (see Supplemental Figure S3, excluding an outlier flux for the glutamate synthesis).

Supplemental Table S3: Predictions of oxygen (O_2) uptake rate and carbon dioxide (CO_2) secretion rate of yeast for different carbon sources.

Experimental measurements are provided by Daran-Lapujade et al. [1]. The relative difference (δ) denotes the absolute value of the difference between the corresponding prediction and experimental measurement divided by the latter. The units for both oxygen (O_2) uptake rate and carbon dioxide (CO_2) secretion rate are mmol/h/gDW, i.e., millimole per hour per gram yeast dry weight.

Carbon sources	O_2 uptake rate (mmol/h/gDW)			CO_2 secretion rate (mmol/h/gDW)		
	Prediction	Experiment	δ	Prediction	Experiment	δ
Glucose	2.68	2.74	0.02	3.23	2.85	0.13
Maltose	2.91	3.05	0.04	3.25	3.05	0.07
Ethanol	6.14	6.87	0.11	2.91	3.26	0.11
Acetate	6.19	7.40	0.16	6.80	7.45	0.09

Supplemental Figure S1: Comparison of the predicted and experimentally determined fluxes in yeast grown with (A) glucose, (B) maltose, (C) ethanol, and (D) acetate.

r represents Pearson correlation coefficient between the predicted and experimentally determined fluxes. mmol/h/gDW, millimole per hour per gram yeast dry weight.

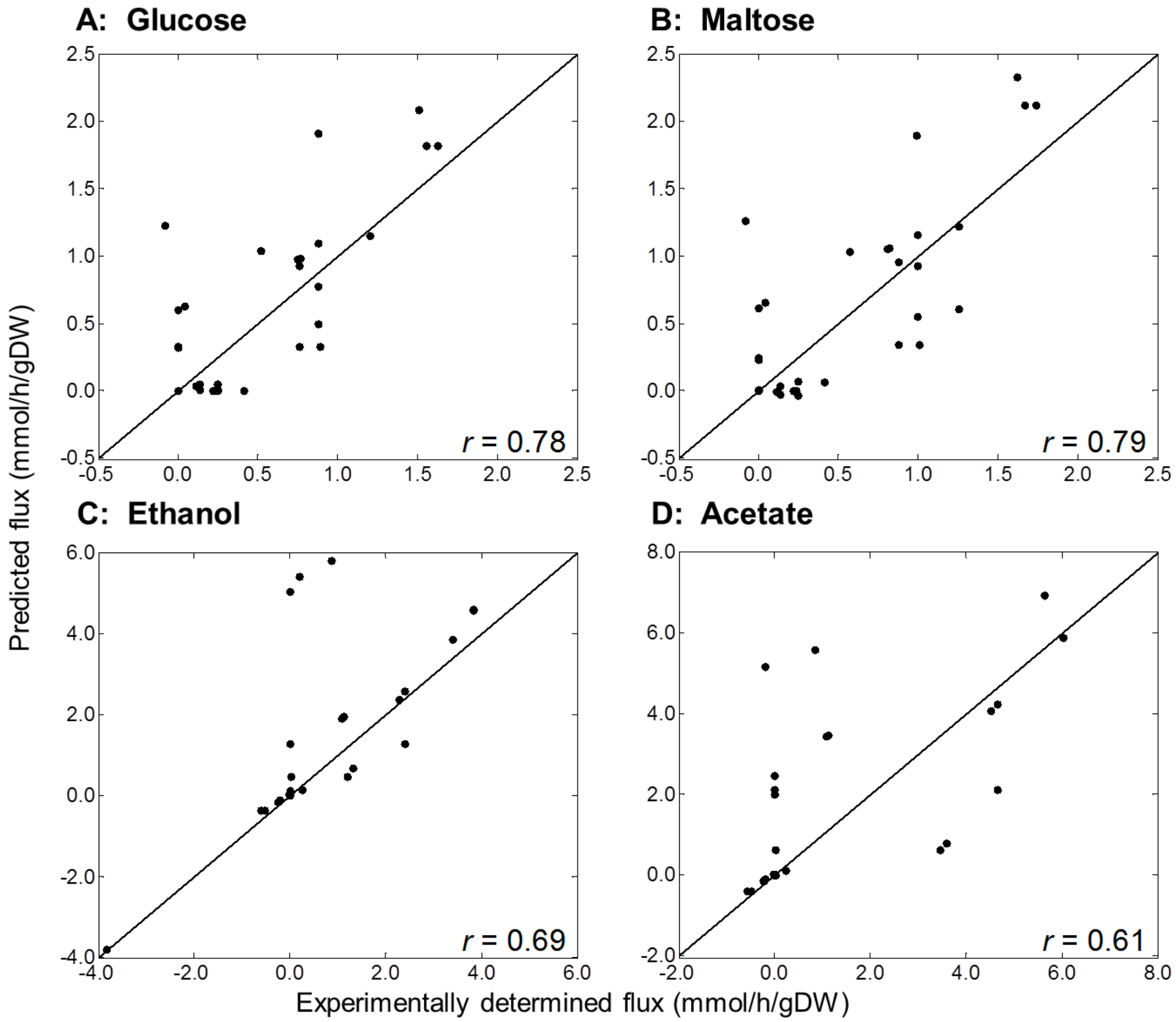

Supplemental Table S4: The predicted and experimentally determined numbers of reaction fluxes that increased, decreased, or exhibited no change.

Based on the difference between the corresponding Δ*gcn4* and wild type fluxes, we classified all reaction fluxes into those which increased, decreased, or exhibited no change if the change was greater than a positive threshold T, smaller than $–T$, or between $–T$ and T, respectively. We constructed the contingency table using the threshold value of $T = 1.3$ (arbitrary units used by Moxley et al. [2]) as an indicator of a significant flux change.

		Experimental flux changes		
		Increase	No change	Decrease
Predicted flux changes	Increase	10	4	6
	No change	4	18	1
	Decrease	4	23	20

Supplemental Figure S2: Robustness of *p*-value estimate from Fisher's exact test for different values of the threshold *T*.

Given a range of threshold values between 0.001 and 1,000, we generated a 3×3 contingency table for each *T* value to gauge the qualitative similarity between the predicted and experimentally measured flux changes as outlined in the caption of Supplemental Table S4. We performed a Fisher's exact test of each table to obtain a corresponding *p*-value, and the graph indicates that a *p*-value estimate of less than 0.05 was robust for thresholds between 0.2 to 100.

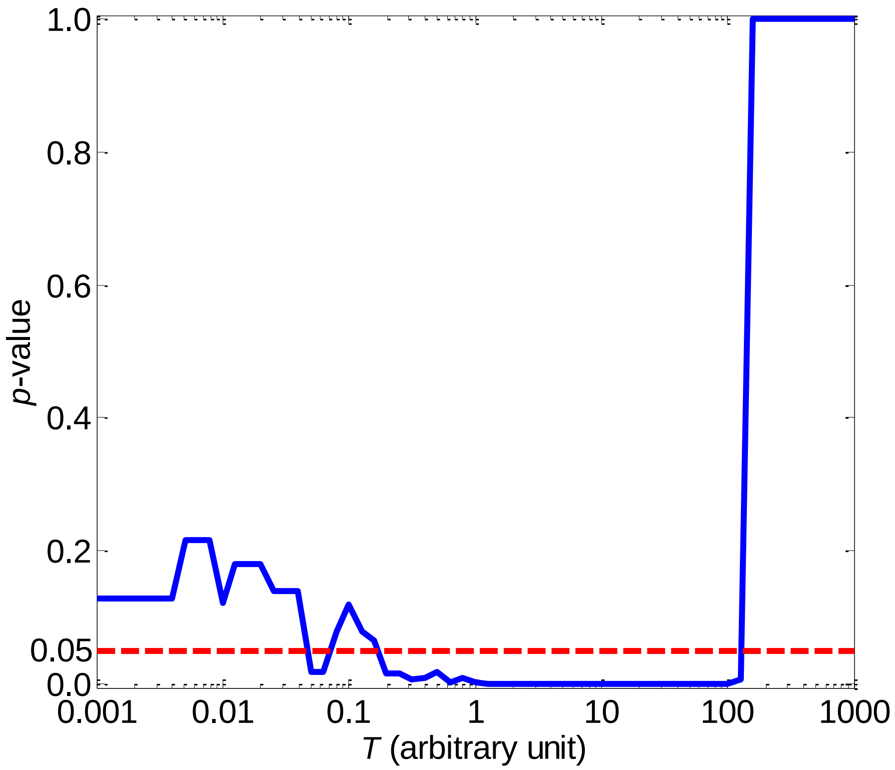

Supplemental Figure S3: Comparison of the predicted and experimentally measured flux differences.

The change in flux (ΔFlux) represents the difference in reaction fluxes between the Δ*gcn4* deletion mutant of yeast and its wild type strain. We compared our computationally predicted ($\Delta Flux_{pre}$) values with the experimentally measured ($\Delta Flux_{exp}$) by Moxley et al. [2] using their high-confidence data set, consisting of 24 fluxes. The flux changes are expressed in arbitrary units. The Pearson correlation coefficient *r* for this selected set of data was 0.79 (excluding an outlier flux for the glutamate synthesis).

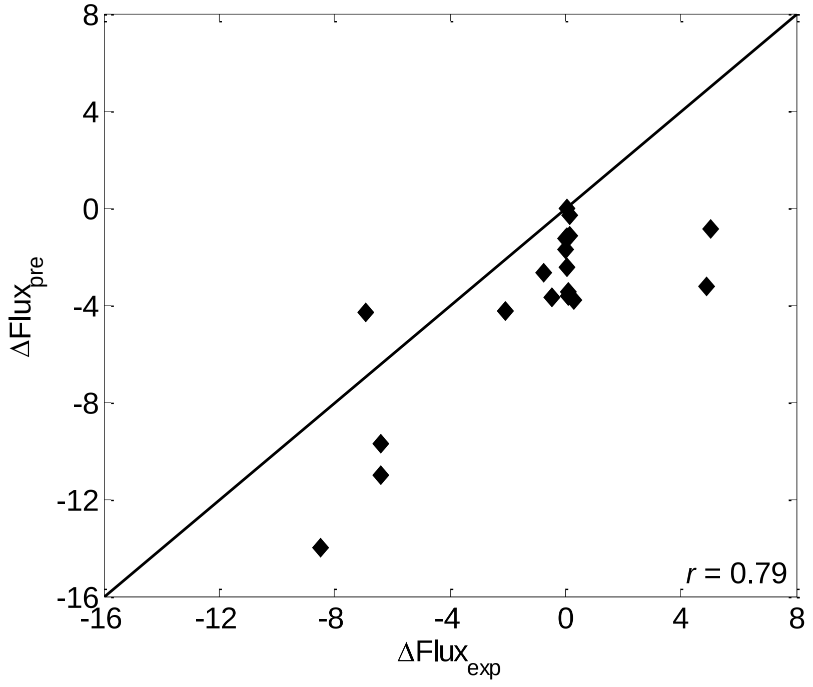

References

1. Daran-Lapujade P, Jansen ML, Daran JM, van Gulik W, de Winde JH, et al. (2004) Role of transcriptional regulation in controlling fluxes in central carbon metabolism of Saccharomyces cerevisiae. A chemostat culture study. J Biol Chem 279: 9125-9138.
2. Moxley JF, Jewett MC, Antoniewicz MR, Villas-Boas SG, Alper H, et al. (2009) Linking high-resolution metabolic flux phenotypes and transcriptional regulation in yeast modulated by the global regulator Gcn4p. Proc Natl Acad Sci U S A 106: 6477-6482.
