# Supplementary material for: Modeling Phenotypic Metabolic Adaptations of Mycobacterium tuberculosis H37Rv under Hypoxia
Source: PLoS Comput Biol. 2012 Sep 13;8(9):e1002688. doi: 10.1371/journal.pcbi.1002688 (PMC3441462; doi:10.1371/journal.pcbi.1002688)
Supplement: Text S2 — Biomass change of M. bovis under slow growth conditions. (PDF) [file pcbi.1002688.s005.pdf]

Supplemental Text S2. Biomass change of *Mycobacterium bovis* under slow growth conditions.

To further test the capability of our modeling approach to predict changes in biomass composition, we predicted the biomass changes of *Mycobacterium bovis* upon transfer from a fast chemostat growth condition [1] to a slow growth condition [2]. In order to model this system, we used the differential gene expression of *M. bovis* between the two growth conditions [3] and the Genome-Scale Metabolic Network of Tuberculosis (GSMN-TB), a metabolic network of *Mycobacterium tuberculosis* that is phylogenetically close to *M. bovis* [1]. The coefficients for DNA, RNA, and protein composition in the biomass objective function of GSMN-TB were taken directly from experimental measurements of the corresponding macromolecules in the fast-growing *M. bovis* [1]. The data in Supplemental Table S5 indicated that for four of the five categories of macromolecules (proteins, RNA, DNA, carbohydrates, and lipids), our predicted changes in biomass compositions were in qualitative agreement, i.e., they had the same directions as experimental measurements [2]. Although our predictions were qualitative in nature, they indicated important metabolite changes, e.g., the slow-growth composition of triacylglycerol (TAG) was higher than in the fast-growth one. This supported the importance of TAG in mycobacteria persisting at slow growth rates, e.g., as shown in *M. tuberculosis* H37Rv persisting under multiple stresses [4] and within human macrophages [5].

Supplemental Table S5: Predictions of the slow-growth induced changes in biomass composition of *Mycobacterium bovis.*

In the upper portion of the table, the biomass composition of each metabolite for fast-growing *Mycobacterium bovis* was taken from the corresponding coefficient in the biomass objective function of the GSMN-TB network, whereas the values for the slow-growing bacterium were predicted via our approach. In the lower portion, we aggregated the metabolites into specific categories to facilitate comparisons with experimental data. “Experiment” indicates the corresponding fast- and slow-growth compositions that were experimentally measured [2], and the entries labeled “Computation” were our predicted values. “Increase” and “Decrease” represent the change of the corresponding biomass composition when *M. bovis* was transferred from the fast-growth to the slow-growth condition. “Consistent” and “Inconsistent” indicated whether our predicted change in the corresponding biomass composition was consistent with the experimental values [2].

Change of each metabolite in biomass composition			
Metabolite	Fast-growth composition	Slow-growth composition	Category of macromolecule
Protein	0.214	0.175	Protein
RNA	0.036	0.032	RNA
DNA	0.022	0.023	DNA
Arabinogalactan-peptidoglycan	0.122	0.123	Carbohydrate
Lipoarabinomannan	0.186	0.195	Carbohydrate
Phosphoethanolamine	0.006	0.005	Lipid
Phosphatidyl-myo-inositol mannosides	0.040	0.032	Lipid
Mycolates	0.086	0.068	Lipid
Triacylglycerol	0.016	0.022	Lipid
Poly-L-glutamate/glutamine	0.035	0.027	
Small molecules	0.050	0.062	

Change for each category of macromolecules in the composition					
Macromolecule		Fast-growth composition	Slow-growth composition	Composition change	Comment
Protein	Computation	0.214	0.175	Decrease	Inconsistent
	Experiment	0.214	0.229	Increase	
RNA	Simulation	0.036	0.032	Decrease	Consistent
	Experiment	0.036	0.013	Decrease	
DNA	Simulation	0.022	0.023	Increase	Consistent
	Experiment	0.022	0.032	Increase	
Carbohydrate	Simulation	0.308	0.319	Increase	Consistent
	Experiment	0.219	0.321	Increase	
Lipid	Simulation	0.148	0.129	Decrease	Consistent
	Experiment	0.448	0.339	Decrease	

References

1. Beste DJ, Hooper T, Stewart G, Bonde B, Avignone-Rossa C, et al. (2007) GSMN-TB: a web-based genome-scale network model of Mycobacterium tuberculosis metabolism. Genome Biol 8: R89.
2. Beste DJ, Peters J, Hooper T, Avignone-Rossa C, Bushell ME, et al. (2005) Compiling a molecular inventory for Mycobacterium bovis BCG at two growth rates: evidence for growth rate-mediated regulation of ribosome biosynthesis and lipid metabolism. J Bacteriol 187: 1677-1684.
3. Beste DJ, Laing E, Bonde B, Avignone-Rossa C, Bushell ME, et al. (2007) Transcriptomic analysis identifies growth rate modulation as a component of the adaptation of mycobacteria to survival inside the macrophage. J Bacteriol 189: 3969-3976.
4. Deb C, Lee CM, Dubey VS, Daniel J, Abomoelak B, et al. (2009) A novel in vitro multiple-stress dormancy model for Mycobacterium tuberculosis generates a lipid-loaded, drug-tolerant, dormant pathogen. PLOS ONE 4: e6077.
5. Daniel J, Maamar H, Deb C, Sirakova TD, Kolattukudy PE (2011) Mycobacterium tuberculosis uses host triacylglycerol to accumulate lipid droplets and acquires a dormancy-like phenotype in lipid-loaded macrophages. PLOS Pathog 7: e1002093.
