# Supplementary material for: Modeling Phenotypic Metabolic Adaptations of Mycobacterium tuberculosis H37Rv under Hypoxia
Source: PLoS Comput Biol. 2012 Sep 13;8(9):e1002688. doi: 10.1371/journal.pcbi.1002688 (PMC3441462; doi:10.1371/journal.pcbi.1002688)
Supplement: Text S3 — Generation of a reference flux distribution in Step I. (PDF) [file pcbi.1002688.s006.pdf]

Supplemental Text S3. Generation of a reference flux distribution in Step I.

In Step I of our approach, we generated a set of reference fluxes representative of an organism at its optimal growth through the following procedure. We first performed a flux variability analysis (FVA) [1,2] to calculate the minimum ($v_{i,\min}$) and maximum ($v_{i,\max}$) fluxes through each reaction i under a flux balance analysis (FBA)-predicted optimal biomass production rate μ_{ref}.

We then obtained a feasible reference flux distribution v_i that was closest to the means of the minimum and maximum fluxes and satisfied all constraints, including stoichiometry, lower and upper bounds for fluxes, and maximum biomass production rate. We did this by solving the following optimization problem:

$$\min_{v_i} \sum_i \left| v_i - (v_{i,\min} + v_{i,\max})/2 \right| \qquad \text{(S1)}$$

$$\text{s.t.} \quad \sum_i S_{mi} \cdot v_i - c_{m,ref}\,\mu = 0 \qquad \text{for each metabolite } m \qquad \text{(S2)}$$

$$lb_i \le v_i \le ub_i \qquad \text{for each reaction } i \qquad \text{(S3)}$$

$$\mu = \mu_{ref}\,. \qquad \text{(S4)}$$

where S_{mi} denotes the stoichiometric coefficient for metabolite m in reaction i, $c_{m,ref}$ represents the original coefficient of this metabolite in the biomass objective function, lb_i and ub_i indicate the lower and upper limits of the flux through reaction i, respectively, μ denotes biomass production rate, and μ_{ref} indicates its optimal value from FBA.

Because we used reaction fluxes normalized by the biomass production rate, we recast the above optimization problem using the reference-normalized fluxes. To obtain these fluxes, we linearly

transformed S1-S4 by dividing the objective function and both sides of all constraints by μ_{ref}. This transformation resulted in the following optimization problem:

$$\min_{x_i} \sum_i \left| x_i - (x_{i,\min} + x_{i,\max})/2 \right| \qquad \text{(S5)}$$

$$\text{s.t.} \quad \sum_i S_{mi} \cdot x_i - c_{m,ref} = 0 \qquad \text{for each metabolite } m \qquad \text{(S6)}$$

$$x_i^L \le x_i \le x_i^U \qquad \text{for each reaction } i \qquad \text{(S7)}$$

$$x_\mu = 1. \qquad \text{(S8)}$$

where x_i, $x_{i,\min}$, $x_{i,\max}$, x_i^L, x_i^U, and x_μ denote the corresponding v_i, $v_{i,\min}$, $v_{i,\max}$, lb_i, ub_i, and μ divided by μ_{ref}, respectively.

The solution to the optimization problem S5-S8 was a distribution of normalized fluxes $x_{i,ref}$ that was close to the means of the normalized minimum ($x_{i,\min}$) and maximum fluxes ($x_{i,\max}$) and, at the same time, satisfied all constraints. Therefore, this distribution was representative of a reference condition, in our case *M. tuberculosis* growth under normoxic conditions, and should be a good starting point for determining alterations in fluxes in the perturbed hypoxic state based on altered gene expressions.

References

1. Mahadevan R, Schilling CH (2003) The effects of alternate optimal solutions in constraint-based genome-scale metabolic models. Metab Eng 5: 264-276.
2. Schellenberger J, Lewis NE, Palsson BO (2011) Elimination of thermodynamically infeasible loops in steady-state metabolic models. Biophys J 100: 544-553.
