# Supplementary material for: Modeling Phenotypic Metabolic Adaptations of Mycobacterium tuberculosis H37Rv under Hypoxia
Source: PLoS Comput Biol. 2012 Sep 13;8(9):e1002688. doi: 10.1371/journal.pcbi.1002688 (PMC3441462; doi:10.1371/journal.pcbi.1002688)
Supplement: Text S4 — Additional implementation details for the constraints given in Step III. (PDF) [file pcbi.1002688.s007.pdf]

Supplemental Text S4. Additional implementation details for the constraints given in Step III.

As outlined in the main text, we implemented the following set of constraints:

$$|x_i| \le (R_i + L_i)\,|x_{i,ref}| \quad \text{reaction } i \text{ with } R_i < 1 \tag{S9}$$

$$|x_i| \ge (R_i - L_i)\,|x_{i,ref}| \quad \text{reaction } i \text{ with } R_i > 1 \tag{S10}$$

$$\sum_i S_{mi} x_i - c_{m,ref}(1 + \alpha_m^+ - \alpha_m^-) = 0 \quad \text{each metabolite } m \tag{S11}$$

$$c_{m,ref}(1 + \alpha_m^+ - \alpha_m^-) \ge c_{\min} \quad \text{each metabolite } m \tag{S12}$$

$$x_i \le U_{i,ref}(1 + \beta_i^+ - \beta_i^-) \quad \text{reaction } i \text{ that is an uptake reaction} \tag{S13}$$

$$x_i^L \le x_i \le x_i^U \quad \text{each reaction } i \tag{S14}$$

We further converted constraints S9 and S10 (constraints 1 and 2 in the main text) into a set of linear inequalities to avoid the absolute values formulation when considering reversible and irreversible reactions. Thus, we imposed constraint S9 via,

$$0 \le x_i \le (R_i + L_i)\,|x_{i,ref}| \quad \text{irreversible reaction } i \text{ with } R_i < 1 \tag{S15}$$

$$-(R_i + L_i)x_{i,ref} \le x_i \le (R_i + L_i)\,|x_{i,ref}| \quad \text{reversible reaction } i \text{ with } R_i < 1 \tag{S16}$$

and S10 via:

$$x_i \ge (R_i - L_i)\,|x_{i,ref}| \quad \text{irreversible reaction } i \text{ with } R_i > 1 \tag{S17}$$

$$-x_i \le -(R_i - L_i)\,|x_{i,ref}| + y_i B \quad \text{reversible reaction } i \text{ with } R_i > 1 \tag{S18}$$

$$x_i \le -(R_i - L_i)\,|x_{i,ref}| + (1 - y_i)B \quad \text{reversible reaction } i \text{ with } R_i > 1 \tag{S19}$$

where B indicates a large number, here set to 10^3, and y_i denotes a binary variable. When y_i was equal to zero, S18 and S19 constrained the value for x_i to be no less than $(R_i - L_i)|x_{i,ref}|$. When y_i

was equal to one, S18 and S19 constrained x_i to be no more than $-(R_i - L_i)|x_{i,ref}|$.

We implemented the adjustment of the biomass composition as an increase or decrease of already exiting components, but we did not address the larger question as to how to allow for a removal or introduction of other metabolites into the biomass objective function. Thus, while we implemented no upper limits, we added constraint S12 (constraint 4 in the main text) to place a lower limit $c_{\min}$ on the coefficients in the biomass function and set it to be 50% of the minimum coefficient in the original biomass function. The subsequent results presented in the paper were robust with respect to this choice, as we varied $c_{\min}$ from 10% to 90% and observed no material difference in our results.

Constraint S14 (constraint 6 in the main text) sets the normalized flux through a non-uptake reaction x_i to be between its lower (x_i^L) and upper bounds (x_i^U). The values for x_i^L and x_i^U were equal to the corresponding original lower and upper bounds in metabolic network divided by the reference optimal biomass production rate.

We used constraints S11–S19 when we solved the optimization problems in Step IV.
