# Supplementary material for: Modeling Phenotypic Metabolic Adaptations of Mycobacterium tuberculosis H37Rv under Hypoxia
Source: PLoS Comput Biol. 2012 Sep 13;8(9):e1002688. doi: 10.1371/journal.pcbi.1002688 (PMC3441462; doi:10.1371/journal.pcbi.1002688)
Supplement: Text S5 — Detailed descriptions for solving the optimization problems outlined in Step IV. (PDF) [file pcbi.1002688.s008.pdf]

Supplemental Text S5. Detailed description for solving the optimization problems in Step IV.

In the first stage of Step IV, we minimized the sum of slack variables $\sum L_i$ subject to constraints S11–S19 in Supplemental Text S4, and obtained the value for $L_{\min}$ as the minimum sum of L_i.

In the second stage of Step IV, we calculated the minimal modification in the biomass objective function by iteratively determining the values for function α_m^+ and α_m^- for each mth constraint in S11. We introduced an intermediate variable $\alpha_{\max}$ that caps the values of α_m^+ and α_m^-. For each iterative step, we first minimized the value of $\alpha_{\max}$ subject to constraints S11–S19 and three additional constraints:

$$\sum L_i \leq L_{\min} \tag{S20}$$

$$0 \leq \alpha_m^+ \leq \alpha_{\max} \quad \text{each metabolite } m \tag{S21}$$

$$0 \leq \alpha_m^- \leq \alpha_{\max} \quad \text{each metabolite } m \tag{S22}$$

where constraint S20 maintains agreement of constraints S9 and S10 in Supplemental Text S4, and constraints S21 and S22 cap the values for α_m^+ and α_m^- at $\alpha_{\max}$. Given the optimal solution for this minimization problem, we then removed the inequalities in constraint S21 for which Karush-Kuhn-Tucker (KKT) multipliers were positive, set the corresponding α_m^+ and α_m^- to be equal to their current solution values, and proceeded to the next iteration. We terminated the iterations once all values for α_m^+ and α_m^- in S11 had been evaluated.

In the third stage of Step IV, we obtained the minimum modification to the uptake rate's upper

limits in a similar manner to the calculation for the biomass objective function. We iteratively determined the values for β_i^+ and β_i^- in constraint 4. We introduced an intermediate variable $\beta_{\max}$ that caps the values of β_i^+ and β_i^-. In each iterative step, we first minimized the value of $\beta_{\max}$ subject to constraints S11–S20 and the following constraint:

$$0 \le \beta_i^+ \le \beta_{\max} \qquad \text{each uptake reaction } i \qquad \text{(S23)}$$

$$0 \le \beta_i^- \le \beta_{\max} \qquad \text{each uptake reaction } i \qquad \text{(S24)}$$

We then used the KKT multipliers from the inequalities in constraints S23 and S24 to determine the values for β_i^+ and β_i^-. We terminated the iterations once all values for β_i^+ and β_i^- in S13 had been evaluated.

In the fourth and final stage of Step IV, we determined the minimum and maximum fluxes for each reaction. For the overall limiting oxygen uptake reaction in hypoxic *M. tuberculosis*, we first calculated the normalized flux through this reaction. We did this by fixing $L_{\min}$, α_m^+, α_m^-, β_i^+, and β_i^- to their previously determined values and minimizing the limiting reaction flux subject to constraints S11–S20. Subsequently, given the determined values for $L_{\min}$, α_m^+, α_m^-, β_i^+, and β_i^- and the limiting oxygen uptake reaction flux, we minimized and maximized the value for x_i for all other reactions and the normalized flux through each reaction i subject to constraints S11–S20.
